# An osteogenic microenvironment improves response during denosumab treatment in giant cell tumors of bone in the spine

**DOI:** 10.3389/fmed.2026.1919109

**Published:** 2026-10-08

**Authors:** Robyn Birch, Felipe Eltit, Tony Ng, Laurence D. Rhines, Ziya L. Gokaslan, Nicolas Dea, Charles G. Fisher, Michael E. Cox, Rizhi Wang, Raphaële Charest-Morin

**Affiliations:** 1 Materials Engineering, University of British Columbia, Vancouver, BC, Canada; 2 Centre for Aging SMART, Vancouver, BC, Canada; 3 Vancouver Prostate Centre, Vancouver, BC, Canada; 4 Department of Pathology and Laboratory Medicine, University of British Columbia, Vancouver, BC, Canada; 5 Department of Neurosurgery, The University of Texas MD Anderson Cancer Center, Houston, TX, United States; 6 Department of Neurosurgery, The Warren Alpert Medical School of Brown University, Providence, RI, United States; 7 Department of Neurosurgery, Rhode Island Hospital, Providence, RI, United States; 8 Combined Neurosurgical and Orthopaedic Spine Program, Vancouver General Hospital, University of British Columbia, Vancouver, BC, Canada; 9 School of Biomedical Engineering, University of British Columbia, Vancouver, BC, Canada

**Keywords:** denosumab, giant cell tumor, mineralization, spine, treatment response

## Abstract

**Introduction:**

Giant cell tumors of bone are benign but locally aggressive tumors. Typically diagnosed in individuals between the ages of 20 and 40 years, these tumors cause significant amounts of pain, and morbidity. Giant cell tumors of bone can be treated with an increased dosing regimen of denosumab, a monoclonal antibody that targets receptor activator of nuclear kappa-B ligand, which has shown promise in reducing pain and tumor size, while contributing to improved ossification of the tumor. However, the ossification response observed varies, with some patients responding rapidly with total tumor ossification, while others respond slowly, if at all. Due to gaps in our current understanding of what improves ossification in patients, we aimed to identify patient specific biomarkers that contributed to increased ossification in our patient cohort.

**Methods:**

Pre-treatment biopsy samples from 19 patients collected from 3 North American centres were grouped based on their response to denosumab, determined from their change in Boriani ossification grade after treatment. The 19 patients pre-treatment tissue was used in gene expression analysis using RNA extraction and nanoString analysis. A subset of tissue from 6 patients and 3 healthy controls were included in protein expression analysis, where differential expression and gene set enrichment analysis was conducted to identify upregulated proteins and pathways contributing to the different treatment responses.

**Results:**

A distinct microenvironment was identified through both genetic and proteomic expression. Good responders demonstrated upregulation of TNFRSF11B and CX3CL1, genes that are related to bone remodeling, and a downregulation of CX3CR1. Additionally, tissue remodeling and cell proliferation markers were upregulated, while immune related markers were downregulated in our good responders (increased ossification of tumor). Genetic analysis was complemented by protein analysis which in our good responders demonstrated upregulation of bone pathways associated with bone development, bone mineralization, ossification, and osteoblast differentiation, and downregulation in bone remodeling, bone resorption, and osteoclast differentiation. Further analysis identified upregulation of LTF, FBLN5, LOX, CHAD, and OGN, and downregulation of CEBPB, CTSK, and TFRC.

**Discussion:**

Giant cell tumors of bone show varied mineralization responses to denosumab, with the biological factors associated with this variability remaining poorly understood. We used gene and protein expression analysis of pre-treatment biopsy tissue to compare good and poor mineralization responses. The good responders demonstrate a pre-treatment tumor microenvironment that favors bone formation and reduced resorption, suggesting that biomarker analysis of biopsied samples may help to understand a patient's response to denosumab and guide personalized treatment strategies.

## Graphical Abstract

**Figure:**
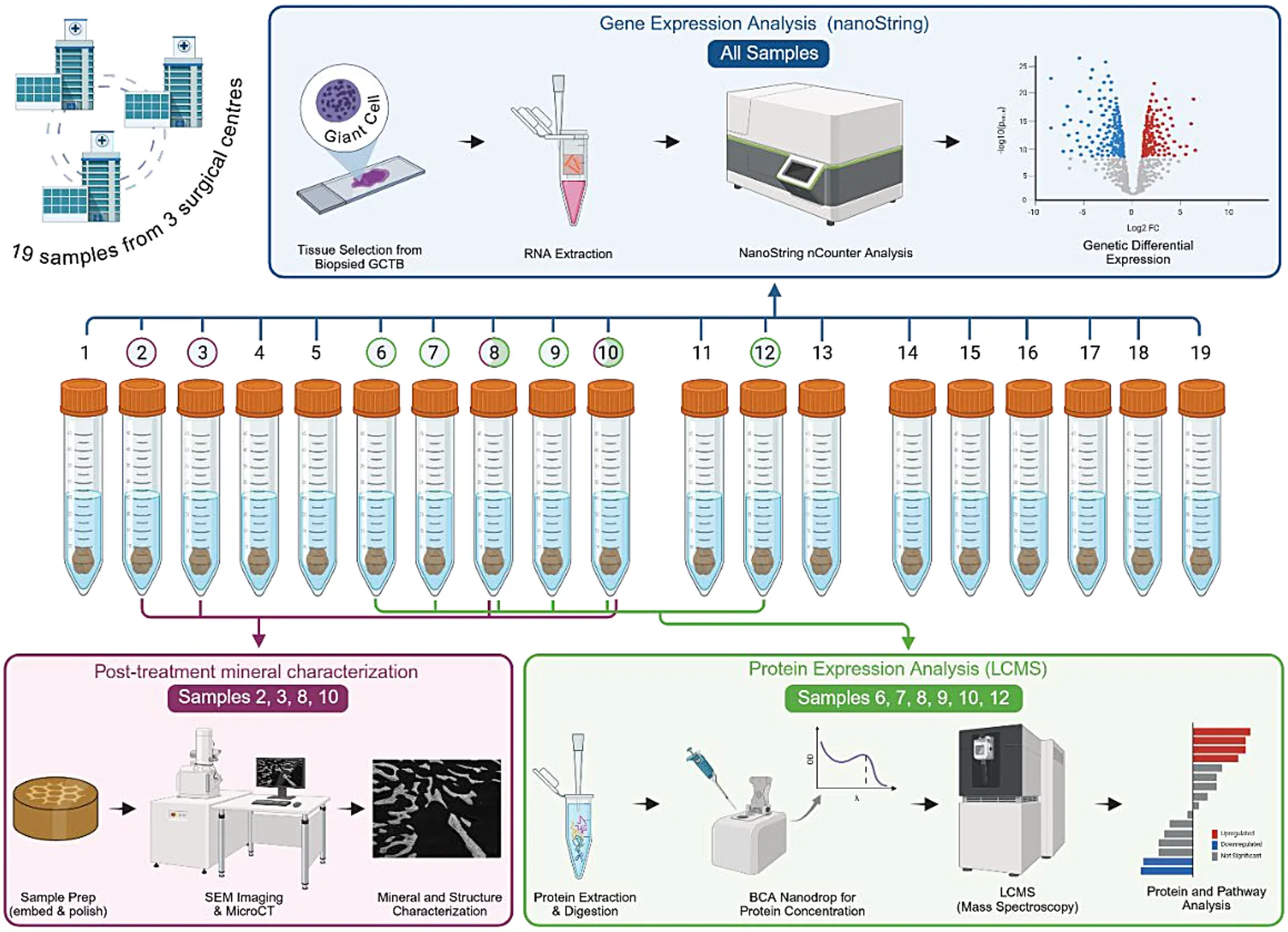

## 1 Introduction

Giant cell tumor of bone (GCTB) is a rare and aggressive tumor characterized by significant osteolysis, which lead to severe pain and pathologic fractures (1). Although benign in nature, in rare cases they can undergo malignant transformation (2). Treatment is surgical resection, with high local recurrence when an intralesional resection is performed. Spinal GCTBs are uncommon and represent no more than 10% of all cases (3–6). Spinal GCTB presents major clinical challenges given their anatomical proximity to critical neural and vascular structures leading to difficulty in achieving optimal surgical margins (7, 8).

Histologically, GCTBs are heterogeneous tumors containing various cell types: multinucleated giant cells, mononuclear spindle-like stromal cells, and monocytes. The key pathogenic factor in GCTBs is their stromal cells, which express and secrete increased amounts of receptor activator of nuclear factor kappa-beta ligand (RANKL, also known as TRANCE, ODF, and OPGL (9) in the tumor (10–12). RANKL and osteoprotegerin (OPG) have been identified as key regulators of bone resorption and osteoclast differentiation (13, 14). RANKL, produced and expressed by the stromal cells, osteoblasts, and osteocytes binds to RANK (15–17), a transmembrane receptor on the osteoclast precursor cells (16), initiating osteoclast maturation, and bone resorption. In normal bone, as it remodels, the osteoblastic cells and osteocytes will release OPG, which acts as a RANKL antagonist, or decoy receptor, blocking RANKL-RANK interactions (15), protecting bone from resorption (13). RANKL is found in higher amounts in osteocytes, and in cases of GCTB and OPG is often unable to offset the difference (18). As a result, several studies have recommended OPG treatments (9) or RANKL inhibitors (19–21) for preventing bone loss and minimizing bone resorption in cases of nonmetastatic tumors like GCT (12).

Since 2013, denosumab has been approved for use in treatment of unresectable and recurrent GCTBs (19–21). Denosumab is a human monoloncal antibody targeting RANKL, inhibiting osteoclast activation through blocking of the RANK-RANKL pathway, acting similar to the naturally occurring OPG (22). Initially denosumab was used in cases of metastatic bone cancer to prevent major skeletal events. Denosumab showed to be an effective therapeutic for increasing bone mineral density (BMD) and lowering bone turnover rates (22), with other areas of research focusing on evaluating the effectiveness of denosumab based on tumor progression, recurrence of disease, and stabilization of the tumor (5, 20, 21, 23). For over a decade, denosumab has been used as a neoadjuvant therapy or stand-alone treatment in GCTB, with reported response rates of 90% (24, 25). Furthermore, denosumab can reduce tumor size (23) and pain symptoms in as many as 97.5% of patients (24), with symptoms resolving in as fast as one month (1). From a surgical perspective, shrinkage and ossification facilitate achievement of desired surgical margins and reduce the anatomical insult (23).

In our previous report, we quantified mineralization in denosumab-treated GCTB compared to normal spinal bone (26). We observed woven bone formation in the treated tumor regions, accompanied by mineralization effects related to treatment durations—specifically, increased mineral content in the mineralized tumor (26). While this quantification of mineralization contributed to our understanding of the degree of mineralization in the tumors post-treatment, a key unresolved question remained: can a patient-specific response to denosumab be identified before it's administration for treatment of giant cell tumors in the spine? Our goal was to determine a relationship between a patient's ossification response and biomarkers detected through genetic and proteomic expression analysis. Based on these biomarkers, we hope to identify patterns present in the tumor to assist in identifying if the patient's response to denosumab can be better understood before treatment begins.

## 2 Experimental methods

### 2.1 Patients

Nineteen patients from three institutions (Vancouver General Hospital, MD Anderson Cancer Center, and Rhode Island Hospital) who underwent neoadjuvant or stand-alone denosumab for spinal GCTB were analysed (Supplementary Table S2, IRB UBC, H22-03461). GCTB samples were acquired for pathological diagnosis of GCTB. An additional three healthy control vertebrae were collected through the UBC Body Donation Program (IRB UBC, H21-02668, Supplementary Table S3). While samples could not be age matched, they were matched for location.

### 2.2 Gene expression analysis

Tissue containing high tumor content (>80%), identified by a trained pathologist, was selected from 19 formalin-fixed paraffin-embedded biopsy specimens acquired prior to denosumab treatment. Using similar methods to Eltit et al. (27), total messenger RNA (mRNA) was isolated from the specimens using a formalin-fixed paraffin-embedded RNA extraction kit (Roche Molecular Diagnostics, Pleasanton, CA, USA) following the manufacturers' instruction. Both RNA purity and concentration were assessed using a microvolume spectrophotometer Nanodrop ND-100 (NanoDrop Technologies, Wilmington, DE, USA). Confirmation of the quality of the extracted RNA was completed using an RNA integrity number between 1.96 and 2.04, and final RNA specimens were diluted to a final concentration of 100 ng/μL. Transcript profiles for 770 genes were determined on an nCounter Digital Analyzer (NanoString, Seattle, WA, USA), using the nCounter PanCancer Immune Profiling Panel (V1.1).

All 19 pre-treatment samples were included in the analysis, with confounding factors including age, sex, response to denosumab after treatment assessment, and batch. Age was grouped into youth, middle-aged, and elderly (25≥, 26–49, and ≥50, respectively). Response to denosumab was determined based on the Boriani grade (23) in three-month intervals during denosumab treatment, as evaluated by the spine surgeon at each independent centre (Supplementary Figure S1). Interobserver agreement was not calculated due to limitations in cross-centre longitudinal image sharing for independent review by all surgeons included on this study. Patients who experienced little to no ossification change (defined by a grade increase less than or equal to 1 at a treatment time of 6 months) were defined as poor responders, with increased changes (a grade increase of 1< at a treatment time of 6 months) defining a good responder. Three patients (samples 5, 8 and 17) had their denosumab treated tumors surgically removed before the 6-month threshold, with their most recent Boriani grade before surgical intervention used for classification*.* Batch refers to the separate batches required to run all samples (1 or 2), with two samples being ran in both batches to confirm batch comparability (Supplemental Methods Section 1.1).

Analysis of the transcript profiles was completed using nSolver Advanced Analysis (v4.0, NanoString, Seattle, WA, USA), normalized using two built-in dynamic normalization methods on mRNA. Positive control normalization used six control probes to calculate a geometric mean normalization factor. CodeSet content normalization was performed using dynamically selected housekeeping genes whose RNA counts and a %CV criteria (CC2D1B, ERCC3, and GPATCH3). The geometric mean was then calculated for selected housekeeping genes to determine sample-specific normalization factors (Supplementary Table S4), which were applied to all genes included in the analysis. Predictors for clustering included age, sex, and response type, with the responder type used as a predictor during differential expression analysis, with the poor responder group used as a baseline reference.

Two patients did not have final Boriani grades and were listed as “Unknown.” When completing differential expression analysis, these samples were relabeled based on their initial clustering to preserve the cohort numbers (Supplementary Figure S2). To evaluate if this reclassification of the two patients with unknown response classifications affected the results, we confirmed that the key exploratory genes and patterns from the differential expression analysis were preserved following their exclusion using a sensitivity analysis outlined in the Supplemental Methods (Section S2.3).

### 2.3 Protein extraction and proteomic analysis

Proteins were extracted from previously processed formalin-fixed paraffin-embedded GCTB pre-treatment biopsies (6 × 5 μm sections) using standardized, established methods (26, 28) (details in Supplemental Methods Section 1.2). Briefly we deparaffinized 5–7 5uM histological sections by a series of xylene washes, followed by sonication. The peptide concentration was measured by Nanodrop. Liquid Chromatography-Mass Spectrometry (LCMS, Easynano 1200, connected to Orbitrap Lumos mass spectrometer) was performed, with a label-free data independent acquisition (DIA), using 5 Da windows for ion selection and HCD fragmentation of ions in that mass range. The search platform was diaNN 1.81 and search parameters include: trypsin digestion, carbamidomethylation, N-terminal M excision and methionine oxidation, peptide length range 7–30, precursor change 1–4, precursor m/z 300–1,800, fragment ion m/z 200–1,800, mass accuracy of 10 ppm, Match Between Runs on (MBR), double pass mode, RT dependent cross-run normalization, with a precursor FDR of 1%.

Proteomics was conducted to compare protein expression between 6 pre-biopsy specimens based on their response type, good or poor, as well as 3 vertebral controls. Protein intensities were then log_2_ transformed and median normalized before being filtered to remove proteins that had more than 75% missing values for a protein (sensitivity analysis on missingness threshold is outlined in Supplemental Methods Section 1.4). The remaining missing values were imputed using a k-nearest neighbors' imputation (*k* = 3) (29).

Due to vertebral controls being processed separately from the GCTB biopsies, only the GCTB biopsy data was used in differential expression (DE) and GSEA analysis. To accommodate proteins found at higher rates in only the GCTB specimens, the data processing described above was repeated for only the GCTB samples. Differential protein expression for the good and poor GCTB biopsies was evaluated using a linear model with empirical Bayes moderation, with the poor response group set as the baseline reference. Results were evaluated using both raw *p*-values, as well as data processed using false discovery rate (FDR) adjusted *p*-values and visualized in volcano plots.

Gene set enrichment analysis (GSEA) was performed on normalized, filtered, imputed data, mapping protein data to genes. Gene sets were obtained from the Molecular Signatures Database (MSigDB), with analysis focused on bone-related biological processes. GSEA was performed on the ranked gene list to determine a normalized enrichment score (NES), *p*-values, and FDR adjusted *p*-value. Enrichment pathways were visualized using an enrichment curve, leading gene bar plots and heatmaps. Finally, a key gene summary was created using an exploratory scoring metric. Genes were ranked using both an absolute score (log_2_FC multiplied by −log10(adj-*p*-value) and the number of pathways the genes appear in to summarize a group of candidate genes associated with the response groups.

### 2.4 Histological staining

Serial, 5 μm thick, formalin-fixed, paraffin embedded sections were prepared on a microtome (Leica RM2235, Leica Biosystems, Illinois, United States), mounted on microscope slides, and subjected to basic hematoxylin and eosin (H&E) staining. Immunohistochemistry was completed using standard protocols (30) (Supplemental Methods, Section 1.5) and incubated in a diaminobenzidine reagent (DAB Substrate Kit, Abcam, United States) for visualization of the immunodetection. All slides were mounted using a mounting medium (Epredia, Fischer Scientific). Microscopic observations were made on an upright brightfield microscope (Carl Zeiss, Germany) and imaged using a cooled camera and ZEN software (Zeiss). All images were background corrected in MATLAB (R2022b, Mathworks) using mathematical image operations previously outlined by Landini (31).

## 3 Results

### 3.1 A larger change in post-treatment Boriani grades is associated with increased microscopic bone volume observed with micro-computed tomography (micro-CT)

Response to denosumab treatment has been described as a deposition of irregular bone structurally compatible with woven bone (26). To confirm that the described response (good or poor) was associated to such a bone deposition, we started our analysis with micro-CT observations on a subset of denosumab-treated tumors from four patients and quantified the bone volume/total volume ratio (BV/TV) of the excised tumor (26). We obtained a wide distribution in the BV/TV value in good responders (63.1% in Sample 10 and 12.175 in Sample 3) and poor responders (28.5% in Sample 8 and 0.99% in Sample 2). Cross-sectional micro-CT imaging identified distinct mineralization patterns between the two groups (good and poor responders, as defined by their change in Boriani grade, Supplementary Table S1). The good responders demonstrated dense intratumoral mineralization, characterized by a granular or diffuse mineral deposition that occupy most of the marrow spaces (Figures 1A,B). In comparison, tumors from patients classified as poor responders demonstrated little to no mineralization, that when present is observed as scattered, heterogeneous mineralization foci or packets (Figures 1C,D and Supplementary Figures S4Bii–iv).

**Figure 1:**
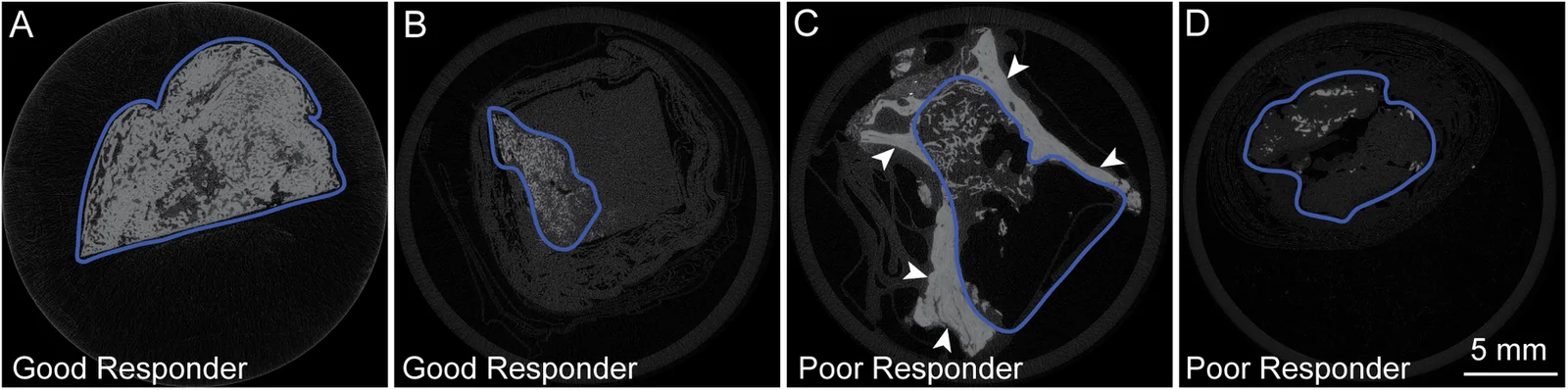
Micro-CT cross-sectional images of four post-denosumab treated tumors with areas of the tumor outlined. **(A,B)** Tumors classified as good responders demonstrate dense mineral packets forming within the tumor region (Samples 10 and 3, respectively). **(A)** A section from the original resected tumor, obtained from the anterior side of the vertebral body, demonstrates extensive dense, homogeneous mineralization. **(B)** Demonstrates a dense packet of mineralization; however, mineralization was not uniform across the tumor region, which was consistent with a comparatively lower BV/TV (26). **(C,D)** Tumors classified as poor responders show a heterogeneous, scattered mineralization (Samples 8 and 2, respectively). Panel **(C)** shows dense mineralization at the periphery (white arrow), corresponding to pre-existing cortical bone unaffected by tumor involvement while the intratumoral regions show soft tissue. **(D)** Demonstrates this phenomenon of random, sparse mineralization more clearly, with little to no border existing, and minimal discontinuous mineralization in the tumor region. Scale bar = 5 mm.

### 3.2 Pre-treatment expression of fractalkine, osteoprotegerin, and fractalkine receptor is associated with clinical prognosis in denosumab treatment of giant cell tumors

Clinical CT images demonstrated varying ossification of tumors regardless of treatment duration, indicating that patient-specific response may contribute to the varied mineralization outcomes observed. We collected pre-treatment biopsies used for clinical diagnosis of GCTB and performed a gene expression analysis by using the Nanostring platform, to identify genes contributing to better mineralization responses during denosumab treatment. We performed unbiased analysis including clinical data, and identified 769 genes (Supplementary Figure S2), of which after QC and low data filtering, 492 genes were used in differential expression (DE) analysis. Thirty-five genes were differentially expressed between good and poor responders, meeting criteria *p*-value < 0.05 and |log_2_FC| > 1 (Figure 2 and Table 1). We found that 15 genes were upregulated in the good responders (Figure 2 and Supplementary Table S7), which corresponded to cellular signalling and regulatory processes, tissue remodelling, and cell growth. We also identified 20 genes that were downregulated in the good responders (Figure 2 and Supplementary Table S7), which corresponded to immune and inflammatory signalling, lymphocyte activation, cytotoxic response, and innate immunity. Additional analysis on individual gene expression is provided in supplemental results (Section 1.6).

**Figure 2:**
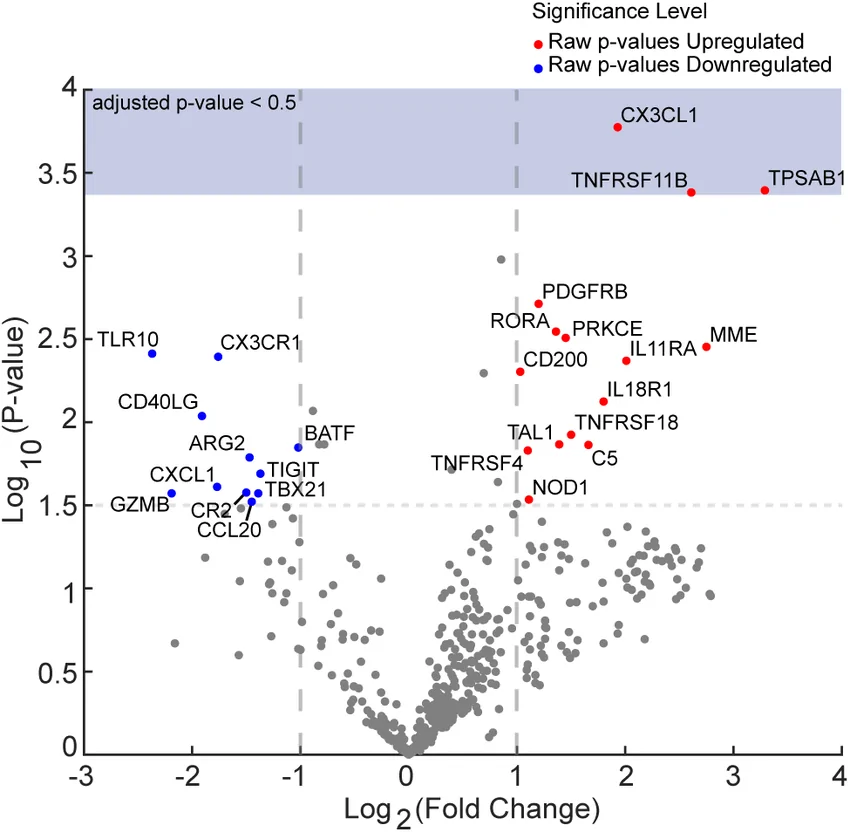
Differential expression with poor responders used as a baseline identifies up- and downregulated genes based on raw *p*-values. The shaded region indicates an adjusted *p*-value threshold of 0.5, which is the lowest significance-indicator threshold displayed by nSolver; however, this remains above the conventional adjusted *p*-value threshold of <0.05. The *p*-values and log2FC values can be seen in Table 1, with the adjusted *p*-values for both the Benjamini–Yekutieli and Benjamini–Hochberg methods listed in Supplementary Table S7, and a full list of genes analyzed in the differential expression analysis provided in Supplementary Table S8.

**Table 1:** Differential expression results for 23 genes in 19 samples that met initial criteria where the raw *p*-value <0.05 and |log2FC| > 1.

Gene Name	Log_2_(FC)	std error (log_2_FC)	Lower confidence limit (log_2_FC)	Upper confidence limit (log_2_FC)	*P*-value	BY *P*-value
TLR10	−2.37	0.685	−3.71	−1.02	0.00387	1
GZMB	−2.19	0.885	−3.92	−0.455	0.0268	1
CD40LG	−1.91	0.632	−3.15	−0.67	0.00918	1
CXCL1	−1.77	0.704	−3.15	−0.394	0.0245	1
CX3CR1	−1.76	0.512	−2.76	−0.754	0.00404	1
SAA1	−1.7	0.732	−3.14	−0.267	0.0356	1
VEGFA	−1.55	0.656	−2.84	−0.266	0.033	1
CR2	−1.5	0.606	−2.69	−0.315	0.0265	1
ARG2	−1.47	0.537	−2.52	−0.413	0.0163	1
CCL20	−1.45	0.599	−2.62	−0.272	0.0301	1
TBX21	−1.39	0.561	−2.49	−0.288	0.0268	1
TIGIT	−1.37	0.526	−2.4	−0.344	0.0204	1
ZAP70	−1.26	0.559	−2.35	−0.162	0.041	1
CYFIP2	−1.13	0.476	−2.06	−0.197	0.0325	1
IL12RB1	−1.07	0.468	−1.99	−0.155	0.0379	1
BATF	−1.02	0.364	−1.73	−0.305	0.0142	1
CD200	1.03	0.309	0.423	1.64	0.00497	1
TNFRSF4	1.1	0.396	0.324	1.88	0.0148	1
NOD1	1.11	0.458	0.215	2.01	0.0292	1
PDGFRB	1.2	0.316	0.582	1.82	0.00194	1
CMKLR1	1.23	0.543	0.167	2.29	0.0397	1
RORA	1.36	0.377	0.622	2.1	0.00285	1
TAL1	1.39	0.493	0.423	2.35	0.0136	1
PRKCE	1.45	0.407	0.653	2.25	0.00311	1
TNFRSF18	1.5	0.52	0.483	2.52	0.0119	1
C5	1.66	0.588	0.505	2.81	0.0137	1
IL18R1	1.8	0.576	0.669	2.93	0.00751	1
ATG16L1	1.83	0.838	0.192	3.48	0.046	1
CX3CL1	1.93	0.38	1.18	2.67	0.000168	0.46
IL11RA	2.01	0.59	0.853	3.16	0.00427	1
CD34	2.02	0.907	0.245	3.8	0.0426	1
ATG10	2.19	0.998	0.234	4.15	0.0455	1
TNFRSF11B	2.61	0.568	1.5	3.72	0.000415	0.46
MME	2.75	0.786	1.21	4.29	0.00352	1
TPSAB1	3.29	0.713	1.89	4.68	0.000403	0.46

A list of the adjusted *p*-values using the Benjamini–Yekutieli (BY) and Benjamini–Hochberg (BH) methods can be seen in Supplementary Table S5, and a full list of all genes identified in differential expression analysis listed in Supplementary Table S6.

We performed pathway analysis by using pathway scores (Figure 3A and Supplementary Figure S5) and Pathview (KEGG Pathways) in nSolver. The pathways with the largest deviation between the two response classifications were: Regulation (mean, standard deviation: 1.323, 5.517 in poor responders vs. −1.470, 0.911 in good responders), Chemokines (1.232, 4.010 in poor vs. −1.375, 0.714 in good), Cytokines (0.956, 2.717 in poor vs. −1.062, 1.042 in good), Interleukins (0.822, 2.082 in poor vs. −0.914, 2.082 in good), and T-Cell Functions (0.641, 3.300 in poor vs. −0.713, 0.813 in good). We also performed cell score analysis, however, these results did not find difference in cell score (Supplementary Figure S6), perhaps due to the noise generated by the hematopoietic environment of bone marrow. The most significant pathway based on the differential expression results was the Cytokine-Cytokine Receptor Pathway (Figure 3B). Within this pathway, we observed genes with higher log_2_ (Fold Change) and *p*-values for TNF Receptor Superfamily Member 11B (*TNFRSF11B*, OPG, 2.61), a regulator of bone remodeling, fractalkine (*CX3CL1*, *1.93)*, a chemokine involved in leukocyte adhesion and migration, and interleukin 18 receptor 1 (*IL18R1*, 1.8) a pro-inflammatory member of IL-1 family (Figure 2). Additionally, Interleukin 11 Receptor Subunit Alpha (*IL11RA*, 2.01), which is known for contributing to mesenchymal cell differentiation and TNF Receptor Superfamily Member 18 (*TNFRSF18*, 1.5), which encodes GITR and is present upon T-cell activation, contributing to immune regulation have higher abundance in the good responders relative to the poor. Within the down regulated genes in this pathway, we found CX3C motif chemokine receptor 1 (*CX3CR1*, −1.76), the receptor of fractalkine, CXC motif chemokine ligand 1 *(CXCL1*, −1.77), a chemokine that promotes inflammation, and neutrophil chemotaxis, *CCL20 a* chemoattractant for immune cells, and CD40 ligand (*CD40LG*, −1.91), a gene critical in the activation of T- and B-cells *to* have lower log_2_(Fold Change) values (in this case down regulation of these genes means increased activity of the pathway). This is accompanied by a decrease in abundance of C-X-C Motif Chemokine 2 (*CXCL2*, −1.26) and C-X-C Motif Chemokine 3 (*CXCL3*, −1.29), which are associated with inflammatory responses, as well as Interleukin 12 Receptor Subunit Beta 1 (*IL12RB1*, −1.07), which is associated with T-cell proliferation and defense response.

**Figure 3:**
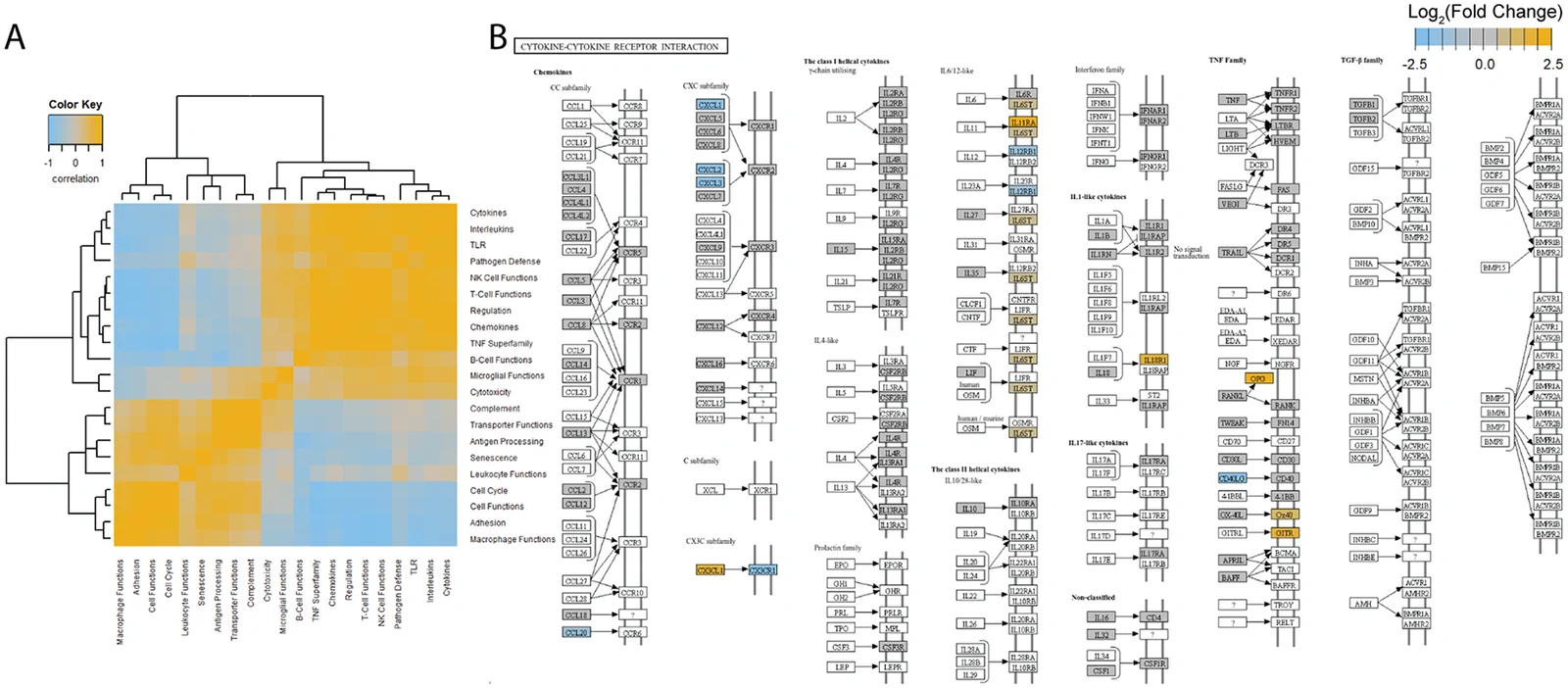
**(A)** A pathway score correlation matrix identifies pathway groups with high positive and high negative correlation. **(B)** KEGG pathway analysis identified the Cytokine-Cytokine Receptor Interaction pathways as the top pathway based on the raw differential expression results. For each KEGG pathway, genes within the panel are mapped to the pathway and the differential expression information is overlaid on the pathway image. Pathway nodes shown in white have no genes in the panel that map to them, nodes in grey have corresponding genes in the panel, however no significant differential expression is observed. Nodes in blue indicate downregulation relative to the selected baseline, and alternatively nodes in orange indicate upregulation relative to the selected baseline.

Combined, these exploratory findings suggest that both good and poor responders may have distinct pretreatment tumor microenvironments that may be associated with their varied mineralization response to denosumab. Good responders demonstrate a tumor microenvironment with enhanced osteoprotective signalling, tissue remodelling, and altered chemokine-mediated communication, based on increased expression of *TPSAB1*, *TNFRSF11B*, and *CX3CL1*. Alternatively, poor responders demonstrate a greater expression of genes associated with inflammatory signalling, immune activation, and lymphocyte-associated responses. While no individual gene remained significantly differentially expressed after multiple-testing correction, the observed nominal changes, combined with the pathway analysis, which provide grounds for further investigation. These differences in gene expression profiles may reflect distinct features of the tumor microenvironment that influence the response to denosumab treatment, potentially contributing to the heterogeneous mineralization observed in GCTB across patients.

### 3.3 Good responders have a microenvironment that promotes bone formation

To confirm the gene expression differences observed and a patients' predisposition to denosumab treatment, we followed our study with proteomics analysis of 6 pre-treatment biopsies. Across both control and GCTB specimens a total of 8,307 proteins were identified. Principal component analysis (PCA) was first performed to assess overall variation in the proteomics dataset, followed by unsupervised clustering of the GCTB biopsy and control samples (Supplementary Figure S8A). These analyses demonstrated a separate clustering of controls from GCTB, confirming the tissue used in proteomic analysis was in fact not bone, but giant cell tumor tissue.

To identify differences between the GCTB good and poor responders, we analyzed the 5,989 proteins identified from the separate proteomics GCTB dataset, which were mapped to their corresponding gene identifiers for downstream GSEA analysis (missingness rates per sample in Supplementary Table S9. We conducted GSEA pathway analysis of bone pathways (Figure 4A). Pathways related to bone formation, osteoblast differentiation and skeletal development were upregulated in good responders, while pathways related to bone resorption, and osteoclast differentiation were downregulated.

**Figure 4:**
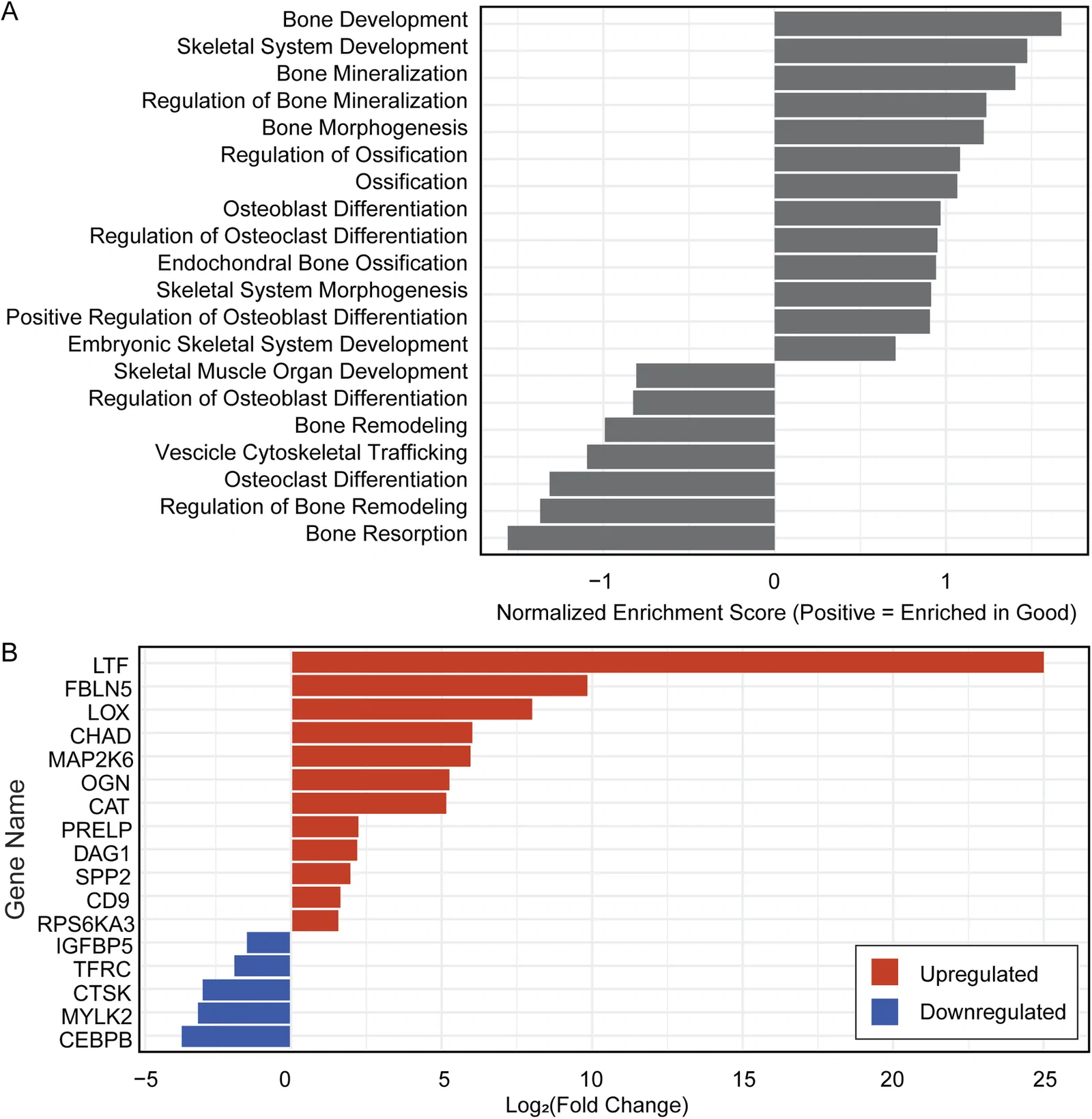
Exploratory pathway analysis for identification of enriched proteins. **(A)** A bar graph illustrating normalized enrichment scores for the top 20 bone pathways. **(B)** Top 17 proteins identified with the highest score when adjusted *p*-value and log_2_ fold-change are assessed (listed by their gene names). This was done to accommodate the low sample size, as adjusted *p*-value approached 1 in the majority of all cases, therefore minimal significance from adjusted *p*-value alone can be made.

Notably, there were several proteins enriched in the good responders when compared to the poor responders. When identifying proteins upregulated in the good responders, we observed proteins that promote bone and skeletal development, osteoblast differentiation and mineralization, and inhibited osteoclastogenesis (Figures 4B, 5A–C). Among the proteins involved in bone development were chondroadherin (CHAD), osteoglycin (OGN), and dual specificity mitogen-activated protein kinase kinase 6 (MAP2K6), and most notably, lactoferrin (LTF), a binding protein with anti-apoptotic effects on osteoblasts and an inhibitor of osteoclastogenesis. Among the proteins involved in the Skeletal System Development pathway are ribosomal protein S6 kinase alpha-3 (RPS6KA3) was identified and is involved in skeletal system development. These were accompanied by ECM structural constituents and assembly markers, prolargin (PRELP), which is involved in anchoring basement membranes to connective tissue, and dystroglycan 1 (DAG1), a complex involved in calcium ion binding. Additionally, fibulin-5 (FBLN5) is associated with elastic fiber formation, positive regulation of osteoblast formation, and calcium ion binding, CD9 antigen (CD9), is responsible for cell adhesion, motility and tumor metastasis, protein-lysine 6-oxidase (LOX) plays a role in osteoblast differentiation and tumor suppression, catalase (CAT), promotes the growth of cells, and sphingosine-1-phosphate phosphatase 2 (SPP2) plays a role in inflammatory signalling. Specifically, when looking at bone pathways, this pattern indicates an environment that promotes osteoblast differentiation and survival, ossification and bone formation, as well as immune and inflammatory response.

**Figure 5:**
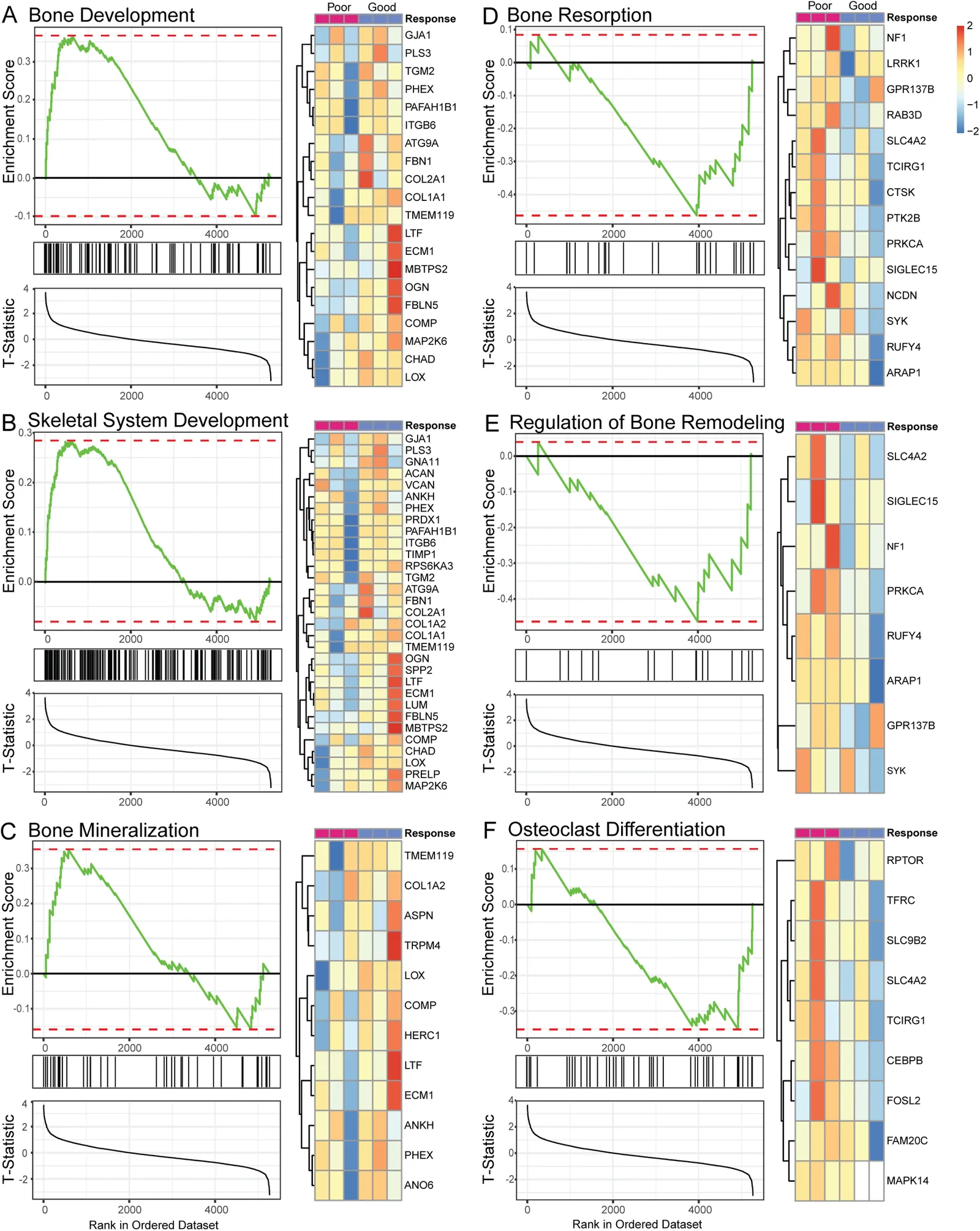
Protein enrichment plots (top) with a T-statistic vs. rank plot (bottom) and a heatmap of the key exploratory proteins in each pathway (right). **(A)** Bone development, **(B)** Skeletal System Development, and **(C)** Bone Mineralization were pathways with the highest enrichment score, indicating higher effects on good responders. **(D)** Bone Resorption, **(E)** regulation of Bone Remodeling, and **(F)** Osteoclast Differentiation were pathways with the lowest enrichment score, indicating higher effects on poor responders. Only leading-edge genes identified during GSEA analysis that were also present in the proteomics dataset were displayed in heatmaps.

Alternatively, downregulated proteins were associated with immunity and inflammatory responses, osteoclast differentiation, and bone resorption and remodeling (Figures 4B, 5D–F). These included insulin-like growth factor-binding protein 5 (IGFBP5), a protein that negatively regulates osteoblast differentiation, transferrin receptor protein 1 (TFRC), a protein responsible for osteoclast differentiation, and cathepsin K (CTSK), a protease involved in osteoclastic bone resorption. There are accompanied by CCAAT/enhancer-binding protein beta (CEBPB), responsible for expression of genes involved in immune and inflammatory responses, and myosin light chain kinase 2 (MYLK2), a kinase involved in skeletal muscle cell differentiation.

When we compared these key proteins to our controls (Figure S8B), it was determined that an overall downregulation of CHAD, LTF, SPP2, IGFBP5, and TFRC in all biopsied samples, indicating reduced bone formation capacity across all samples. This was accompanied by upregulation of OGN, PRELP, LOX, and DAG1 in our good responders compared to controls, indicating good responders may have improved tumor suppression and support from extracellular proteins for skeletal development, whereas the increased bone resorption due to GCTB is supported by an increase in CTSK, a protease that is involved in degradation of collagen type I. These findings indicate that improved denosumab response in GCTB may be associated with upregulation of proteins that support integrity of the extracellular matrix, bone development, and mineralization processes. However, other proteins, including those regulating osteoclast activity and bone resorption are downregulated.

### 3.4 Independent validation using IHC spatially identifies presence of proteins that promote bone synthesis and mineralization

Our previous analysis shows that GCTs that have a better response to denosumab treatment have -prior to treatment- a transcriptomics and proteomics pattern that suggest the presence of a more osteogenic environment. These results suggest that intralesional characteristics determine the therapeutic prognosis. To confirm that this osteogenic microenvironment is localized in the GCT tumor we next performed a histopathological analysis of the specimens (Figure 6). Hematoxylin and eosin staining shows the traditional description of abundant giant multinucleated cells, in a matrix of stromal cells and profuse hemorrhage. We followed the analysis with immunohistochemistry for markers of osteoblastic activity. We observed that good responders showed a stronger staining with a broad distribution of osteocalcin (OCN). OCN is the most abundant non-collagenous protein in bone (32) and is critical for bones' mineral composition and crystal orientation. Good responders also showed a higher immunodetection of Runt-related transcription factor 2 (RUNX2), which expression supress pre-osteoblasts proliferation, inducing the cells towards differentiation into osteoblasts (33). We also observed a stronger immunodetection for osteoprotegerin (OPG) in good responders, which aligns with a more osteoprotective environment prior to denosumab treatment. All sections show positive immunodetection for collagen I (COL1, Supplementary Figure S10), which was observed mostly in the trabecular and irregular bone. Similarly, all samples (good and poor responders) showed a positive stain for OCN (Figure 6 and Supplementary Figure S10), a protein that requires RUNX2 for its expression and is related to osteoblasts differentiation (34). No immunostaining was observed for CXCL1, CTSK and TRAP (Supplementary Figure S10). Combined our results are consistent with the idea that there is a more osteoprotective microenvironment in some GCTBs, which determines a better response to denosumab therapy.

**Figure 6:**
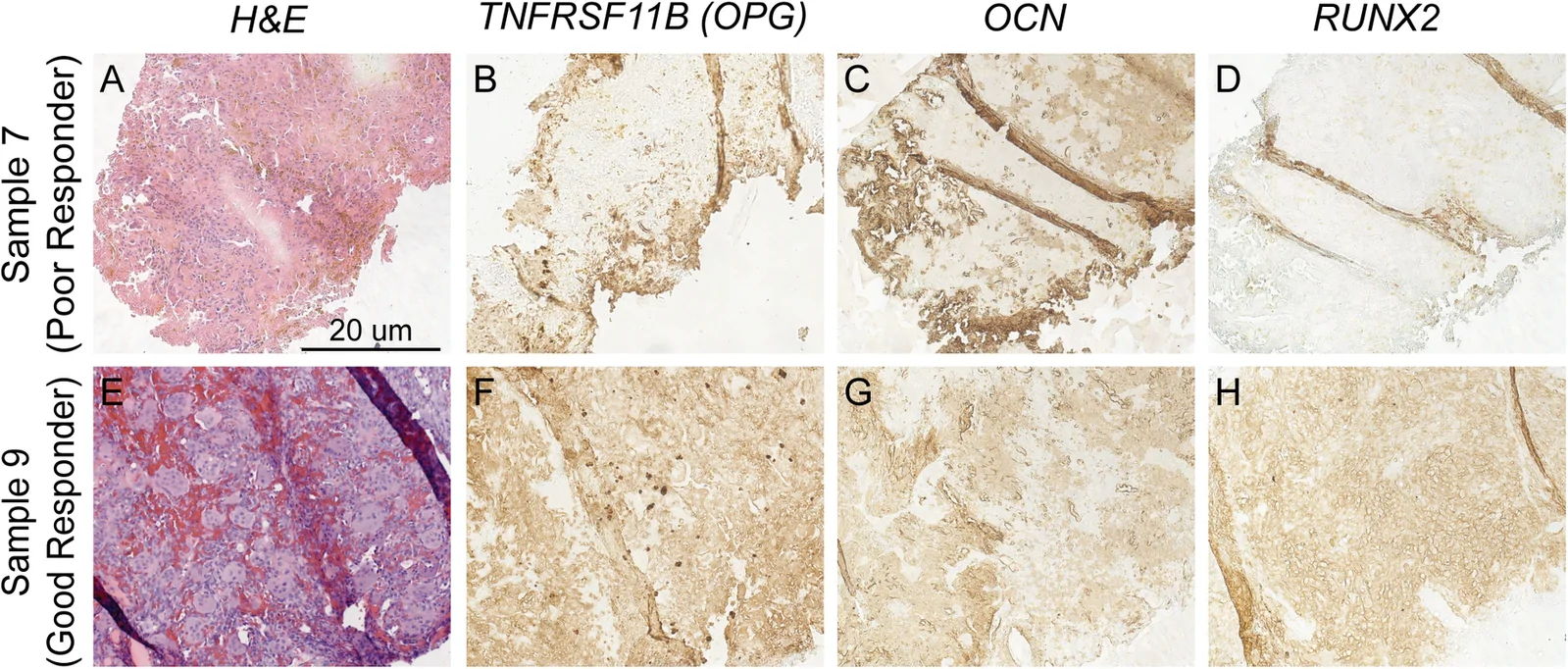
Histopathological brightfield imaging of GCTBs. A-D) H&E staining of poor **(A)** and good **(E)** responders to Denosumab treatment. Both specimens are characterized by the presence of giant multinucleated cells in a matrix of stromal cells and evident hemorrhagic spots. Staining for OPG **(B,F)**, OCN **(C,G)** and RUNX2 **(D,H)** shows a weak scattered immunoreaction in poor responders and a stronger homogeneous staining in good responders. Scale = 20 μm, magnification at 20×. Supplementary Figure S9 illustrates all IHC results at 2.5×.

## 4 Discussion

Denosumab has shown promising clinical benefit in promoting GCTB mineralization before surgery (35). However, the degree of mineralization achieved varies between patients, and the biological factors influencing this variability remain poorly understood. Here, through use of gene expression analysis, proteomics and histological methods, we describe a molecular characterization of GCTBs based on their response to denosumab treatment. Our results support the hypothesis that differences exist between GCTBs that determine their responsiveness to treatment. GCTBs with a good response have a more osteogenic and less osteolytic signature than the poor responding. Despite our limited numbers, our results are consistent through a systematic multimodal analysis, and as such, could help in determining better diagnostics or classification of GCTB's, that may ultimately lead to the establishment of differential treatments based on molecular characterization of a patients tumor.

The RANKL/RANK/OPG pathway is recognized as playing a central role in the osteolytic behavior of GCTB. Studies have shown increased RANKL expression in GCTBs compared to normal bone tissues (12, 36), along with lower OPG levels in GCTB tissue than in normal bone, more evident in recurrent GCTBs (36), supporting the idea of a pre-treatment microenvironment that favors osteoclastogenesis and bone resorption. Our finding of higher TNFRSF11B expression in good responders is notable, as TNFRSF11B encodes OPG. OPG acts as a decoy receptor, inhibiting osteoclast maturation through RANKL-RANK binding—much like the monoclonal antibody, denosumab. Overall, our results suggest the synergistic effects of denosumab, an anti-RANKL antibody, with OPG, the soluble receptor of RANKL, and perhaps pre-treatment quantification of OPG may be an informative response to this therapy. Another interesting result was the upregulated CX3CL1 (fractalkine) expression in good responders, while its receptor CX3CR1 was downregulated. In bone, Kuboi et al. (2022) demonstrated that osteoclast precursors express CX3CR1 (37). Furthermore, they showed that the osteoclastic differentiation induced by RANKL is strongly reduced in CX3CR1 null mice, demonstrating the essential role of this molecule in bone resorption (37). This is concordant with our findings in which patients with low CX3CR1 expression showed a better response to denosumab treatment, observed as a reduced bone resorption and increased bone formation. Fractalkine is a dual-function molecule with a transmembrane molecule found on the cell surface, facilitating cell adhesion and migration (38). Alternatively, fractalkine can be cleaved and the released form functions as a soluble chemokine. In cases of bone metastatic lung cancer, the anti- or pro-tumoral effects of fractalkine have seemed to depend specifically on the tumor characteristics, however it has been shown that increased fractalkine expression reduced bone tumor burdens and reduced the tumors invasive potential (39). A decrease in fractalkine levels appears to also contribute to increased inflammatory monocytes (39), indicating a higher inflammatory response, consistent with the increased inflammatory and immune markers seen in our poor responders. Moreover, previous literature has recognized the anti-resorptive/pro-tumoral effects of CX3CR1 deficiencies, resulting in reduced RANKL as well as other osteoclastic markers (40). Nonetheless, higher TNFRSF11B expression coupled with an imbalance in the fractalkine-CX3CR1 expression levels may reflect an intrinsic reduction of osteoclast activity, producing an unfavorable resorptive environment, which when combined with denosumab, enhances the effects of the treatment.

Our proteomic analysis of pre-treatment biopsies demonstrated enriched proteins involved in bone development and osteoblast differentiation, while suppressing bone resorption and osteoclast differentiation. GSEA analysis based on the protein expression revealed that leading markers including but not limited to LTF, FBLN5, LOX, and CHAD were upregulated in the good responders, while CEBPB and CTSK were downregulated. Furthermore, we found two iron binding proteins, LTF and transferrin receptor, were up- and downregulated in good responders, respectively. This finds empirical support given the fact that LTF has a role in bone regeneration, promoting proliferation and differentiation of osteoblasts, while simultaneously inhibiting osteoclasts (41–43) and acting as an osteoblast survival factor by reducing osteoblast apoptosis (44) and promoting osteoblast differentiation (45). Furthermore, supplemental LTF has been associated with increases in bone mineralization (44, 45), or inversely described to prevent bone resorption (46), increasing the area of mineralized bone observed (39–41). In line with this mineralization-associated environment, FBLN5 was also detected in good responders. FBLN5 is an extracellular matrix protein that plays roles in the formation of matrix scaffolds, fibrous matrices, and elastic fibers (47, 48) and has been shown to contribute to facial skeletal development and premaxillary bone formation (47). When supplemented with FBLN5, improvements to the bone volume to total volume ratio (BV/TV), trabecular number (Tb.N), and a reduction of trabecular separation (Tb.Sp) have been observed (48). Another ECM protein, LOX, contributes to the regulation of bone remodeling and fracture healing, and is found in collagen during bone formation (49). Moreover, the ECM protein CHAD has been described as having anti-resorptive properties and exhibits anti-tumoral effects in cases of cancer-induced bone metastasis (50). In addition to its protective effects, it has also been associated with a higher BV/TV when cyclicCHAD (a peptide derived from the integrin-binding domain of CHAD) is administered (50). Alternatively, CEBPB was the most downregulated in our good responders, indicating higher expression in the poor responders. CEBPB has been previously observed in GCTBs and is known to contribute to the formation of larger osteoclasts with more nuclei (51) and increased RANKL expression (52), suggesting the poor responders may have more aggressive resorptive activity due to larger osteoclasts, accompanied by increased osteoclast differentiation potential. Moreover, CTSK was downregulated in all GCTB samples when compared to our healthy controls but had the lowest expression in poor responders (Figure S5C) (49). CTSK is a primary collagenase that is present upon bone resorption and degradation of the bone matrix (53) and is present in the early stages of osteoclastogenesis (54). CTSK is associated with the activation of RANK (54), is related to the formation of osteoclast-like-cells (54), and has previously been associated with osteoclast cells in GCTBs (55). The higher expression in poor responders may be due to higher presence of osteoclasts in these tumors, increasing bone resorption in the poor responder group. Together, the gene and protein expression findings support the idea of a pre-treatment tumor microenvironment that is more favourable to bone formation and mineralization in patients who will later have an increased tumor ossification during denosumab treatment and described as good responders.

Although we observed differing tumor microenvironments between good and poor responders, this study was a preliminary exploratory analysis of the biological mechanisms involved in GCTBs during denosumab treatment. As expected for a rare disease, the main limitations of our study are the sample size and the lack of complete matched pre- and post-surgical cohorts. The use of biopsied samples also limited the depth of RNA and protein analysis, especially for soluble proteins that may be lost during tissue processing, which may explain why RNA-based inflammatory markers or signalling molecules did not always correspond to the proteomic findings. Additionally, the absence of control samples in the gene expression analysis meant the differential expression was performed with the poor responder group as a baseline. Although healthy controls were included in the protein analysis, separate batching may have affected protein detection between groups. All tumor biopsies used for molecular analysis were collected prior to initiation of denosumab treatment and therefore represent the pre-treatment tumor microenvironment. Treatment duration varied across patients, and while some patients demonstrated continued improvement in lesion grade after the 6-month threshold, this subsequent variation in treatment duration could not influence the pre-treatment molecular profiles analyzed in this study resulting from the established 6-month threshold for classification of treatment response, providing a standardized time point for comparison. Additionally, due to the nature of GCTB, their presentation, and treatment, some patients had lesions with higher grades (increased ossification) upon initial imaging, prior to denosumab treatment. This baseline variation may contribute to differences in subsequent mineralization response, however, could not be controlled for given the rarity and clinical heterogeneity of spinal GCTB and therefore remains a limitation of this study.

In conclusion, our findings suggest that good and poor responders may have different tumor microenvironments before denosumab treatment, and these specific microenvironment differences are associated with their varied mineralization responses to denosumab. Good responders exhibited a profile characterized by osteoprotective signalling, tissue remodelling, and altered chemokine-mediated communication, and proteins associated with bone development and osteoblast activity. In contrast poor responders showed greater expression of genes associated with immune activation and inflammatory signalling, and proteins associated with osteoclast activity and bone resorption. Key exploratory biomarkers upregulated in good responders included but are not limited to *TNFRSF11B*, *CX3CL1*, LTF, FBLN5, LOX, CHAD, and downregulation of *CX3CR1*, CEBPB, and CTSK. Future multicentre studies with a larger cohort of GCTB patients are needed to validate these findings and better determine how histopathological and molecular characterization of GCTBs, treatment duration, dosing strategy, and tumor ossification relate to a patient-specific denosumab response.

## Data Availability

The authors acknowledge that the data presented in this study must be deposited and made publicly available in an acceptable repository, prior to publication. Frontiers cannot accept a manuscript that does not adhere to our open data policies.
