# Supplementary material for: An osteogenic microenvironment improves response during denosumab treatment in giant cell tumors of bone in the spine
Source: Front Med (Lausanne). 2026 Oct 8;13:1919109. doi: 10.3389/fmed.2026.1919109 (PMC13686836; doi:10.3389/fmed.2026.1919109)
Supplement: Supplementary file 1 [file Datasheet1.docx]

# 1 Supplemental Methods

# 1.1 Confirming Batch Comparability in NanoString Analysis

A total of 21 NanoString assays were completed on a total of 19 patient samples, with two samples run in duplicate to assess inter-batch comparability. When initially processing the full dataset, all 21 samples, in addition to the blank control, were normalized together. Based on the normalized gene counts, both Pearson correlation and gene-wise log_2_ fold change (log_2_FC) were assessed to determine if any significant inter-batch technical variations existed between the two batches.

Samples 1 and 5 were the duplicate samples, both showing high concordance between batches, with Pearson correlation coefficients of 0.992 and 0.995, respectively. For each duplicate sample, most genes showed minimal variation between batches, with average absolute log_2_FC of 0.355 and 0.315 and median absolute log_2_FC of 0.289 and 0.213, respectively. When assessing individual genes absolute log_2_FC values, between 4 and 5% of the genes had values above an absolute of 1, and only 0.13% had values above 2. Given the expected inter-tumoral heterogeneity and biological variability within tumor sections, these results supported the comparability of the two batches and justified all further downstream analysis.

# 1.2 Detailed Proteomic Data Collection

The tissue was deparaffinized in a series of two xylene washes. The tissue was then dried in a Centrivap for 15 minutes. 200μL of an extraction buffer containing 100mM Tris pH8, 1mM MgCl_2_, 1mM 3% SDS, 1mM 1% Triton X100, 1mM 1% NP40, 2.5mM EDTA, 2mM DTT was added to each Eppendorf tube. The specimens were then incubated at 85°C for 120 minutes at 1400rpm. The tubes were cooled and then probe tip sonicated for 60 seconds each at 50% (2-4 Watts, ~85 Joules). The specimens were centrifuged at 20000g and a small aliquot (20 uL) was obtained for protein quantification analysis (BCA, see Supplemental Methods Section 1.3). Then 8μL of dithiothreitol (200mM DTT) were added to each specimen and incubated at 65°C for 45 minutes at 1400rpm. The specimens were cooled and then 9μL of iodoacetamide was added to ensure stability of the proteins and peptides. The specimens were then incubated again at room temperature in the dark at 800rpm. Another 18μL of DTT were added to the specimens and incubated at room temperature for 15 minutes. The specimens were then placed into a freezer at -20°C overnight.

A magnetic bead solution (320μL) was prepared and placed on a magnet rack for surfactant removal. The magnet beads were rinsed with 400μL of water twice with surfactant removal after each step. Another 130μL of water was added to the beads and vortexed. Between 10 and 20μL of bead solution was added to each specimen based on their previous BCA results. A 580μL solution of acetonitrile was added to each specimen and incubated at room temperature for 10 minutes at 800rpm. Specimens were then placed on the magnet rack and rinsed with 80% ethanol three times. Then 100μL of Hepes (50mM, pH8) is added to the specimens, followed by 6μL of trypsin/Lys-C (0.4μg/μL) to submerge the beads and tissue bits. The specimens were then incubated overnight at 37°C at 1400rpm.

After overnight incubation, the specimens were placed on the magnet rack, and solution was captured and placed in a new Eppendorf tube. A 2μL surfactant was transferred to a new Eppendorf for BCA of peptides, and the remaining surfactant was placed in the centrifuge for 5 minutes at 20000g. Then 200μL of 0.5% Trifluoroacetic Acid (TFA) was added to the centrifuged specimens. In the SepPak column, 1 mL of 0.1% TFA/acetonitrile (ACN) was added to condition the tube. After 1 mL of 0.1% TFA/water was added to equilibrate the tubes. Then the acidified specimens were added. Then a 1mL 0.1% FA/water wash was completed. Eppendorf tubes were placed under each column and then 0.8mL of 70% ACN/0.1% FA elution was added and ran until the solution was filtered. The specimens were then dried in the Centrivap until dry. After the specimens were dry, the residue was resuspended in 50μL 0.1% TFA. After resuspension, the specimens were incubated at room temperature for 35 minutes at 1400rpm. BCA was then completed again using 1μL of each resuspended specimen to assess the SPE concentration. Then 0.5μg/μL aliquots from each specimen (Table S5) were added to the sample plate and LCMS was conducted as stated in the main text.

# 1.3 Bicinchoninic Acid (BCA) Assay

A BCA protein assay was prepared using a 50:1 ratio of Reagent A to a 40 mg/mL CuCl_2_ in water, equivalent to Reagent B (Pierce Protein Assay, Product #23228). To make standard calibration samples, 38μL of the buffer was mixed with 2μL of each protein standard solution at known protein concentrations (0, 0.1, 0.4, 1, 2, and 5 mg/mL cal). Then 38μL of buffer was mixed with 1-2μL of each sample. The solutions were then incubated at 37°C for 30 minutes. Using a Nanodrop 2000, and BCA Nanodrop software, the standards and samples were measured. The Nanodrop was initially blanked with water prior to sample measurement. The reference concentrations were set and measured in triplicates and data exported to create a quadratic curve of best fit. The samples were measured in triplicate, with remeasurement performed if the concentration deviated $\pm$ 0.005. All sample data was exported for protein concentration assessment.

## 1.4 Sensitivity analysis of proteomic missingness threshold determines most enriched pathways remain consistent.

The missingness value used for k-Nearest Neighbor (kNN) imputation is well accepted, as is the k-nearest neighbors method. However, due to our small cohort remaining after nanoString analysis, a sample size of 6 may introduce bias when imputed with standard missingness thresholds. The standard missingness typically used for kNN=3 in our analysis is 0.75. With a sample size of 6, this means that any protein with 2 or more proteins present will be imputed (i.e. 4 missing will still be imputed). To determine if our pathway analysis and the conclusions based on this analysis were on observations or imputed data, we sought to complete sensitivity analysis with varied missingness threshold values (0.75, 0.5, and 0.25) with 0.25 being the most conservative.

We identified that the three pathways with the lowest score remain the same with a missingness threshold of 0.25. However, at a missingness threshold of 0.5, the Regulation of Bone Remodeling pathway does not appear in the top twenty list based on normalized enrichment scores (NES), however the Osteoclast Differentiation becomes the second lowest NES, with the Bone Remodeling moving to the third lowest NES values. As for the pathways upregulated in this exploratory sample set, the top three pathways remain unchanged, regardless of the missingness threshold used to analyze the proteomics datasets. We the authors do recognize that some of the normalized enrichment scores do change as the missingness thresholds are adjusted, however, we confirm only three pathway changes exist for each of the top twenty pathways as seen in Table S6.

# 1.5 Histological Staining Techniques

## 1.5.1 Procedure for Immunohistochemical Staining

Procedure for Preparing Slides:

1. Prepare HCl solution. Using a pipette, mix 0.833 mL of 37% HCl with 100 mL of distilled water.
2. Select slides to be stained.
3. Using the water bath, heat water to 37°C - 40°C.
4. Place slides in a glass slide holder.
   a. Add 50 mL of HCl solution
   b. Leave for 10 minutes in water bath.
5. Prepare pepsin solution.
   a. To make a 2% pepsin solution, mix 1g of pepsin with the remaining 50mL HCl solution.
   b. Stir with magnetic stirrer on low heat for 5 minutes.
6. After 10 minutes in HCl, move slides into a new slide holder containing the pepsin solution. Leave sitting in the water bath for 20 minutes, remove slides and slightly air dry for 5 minutes.

***Note:*** This process is done to help “hydrate” the proteins in the samples and is done on fixed tissues.

Procedure for Quenching and Blocking:

1. Using Immun-pen, draw a circle two times thick around the sections on the slide (there is normally two or three per slide). ***Note:*** This creates a hydrophobic barrier around each of the sections.
2. Using peroxidase, place drops within each circle to quench the samples, ensuring all tissue is covered. Leave this for 10-15 minutes. ***Note:*** All living tissues contain peroxidase, we must remove it before adding antibodies to the tissue.
3. Label the top of the slide with the appropriate antibodies to be placed on each section.
4. Rinse the peroxidase in a series of PBS rinses.
   a. Turn on vacuum and add a pipette tip.
   b. Collect the drop bottle PBS.
   c. Remove peroxidase and cover tissue in PBS.
   d. Repeat steps b and c for all sections on all slides.
   e. Repeat steps c and d, removing PBS, until a total of three washes have been completed.

***Note:*** Leave each PBS rinse for about 5 minutes each.

5. Prepare primary antibodies.
   a. Retrieve antibodies needed and BSA from the fridge/freezer.
   b. Thaw both the BSA (blood serum albumin) and the antibodies.
   c. Using vacuum, remove PBS and add 50 μL (approximately 2 drops) of BSA using a pipette. Leave this for 10 minutes so the proteins in the albumin have time to interact with the sectioned tissues.
   d. Retrieve as many antibody mixing vials as required and label the lids with the antibody in use.
   e. Add antibody to BSA into the mixing vials and flush pipette tip. Concentrations used in this study can be found in Supplemental Table S1.
   f. Vortex for a few seconds.
   g. Using vacuum, remove BSA and add a minimum of 75 μL (approximately 2-3 drops) of antibody solution using a pipette. If it is less than this but a smaller section this is okay, however ensure total coverage of the sample.
      i. If the sample is to be a negative control, leave the BSA on overnight.
   h. Place all the slides in the humidity chamber and place in the fridge overnight.
      i. The humidity chamber might need to have paper towel added and dampened. Also check that the paper towel is not moldy.
      ii. If needed, add some extra water to the humidity chamber as well as the damp paper towel.
      iii. Alternative incubation processes include:
           - 37°C for 2 hours duration

Procedure for Staining:

1. Remove the humidity chamber from the fridge.
2. Using a vacuum and PBS, wash the sections three times for 5 minutes each.
3. Prepare the secondary antibodies.
   a. Check the type of host the antibody was cultured in (rabbit or mouse).
   b. Retrieve the BSA from the freezer and thaw.
   c. Add a 1:1000 ratio of antibody to BSA into the mixing vials and flush pipette tip.
   d. Vortex for a few seconds.
   e. Once all the secondary antibodies are mixed, place the respective secondary antibodies (~75 μL) on each section, vacuuming the PBS solution from the section.
   f. Leave to incubate at room temperature for 1 hour in the incubation chamber.
4. Prepare the IHC stain.
   a. Using a slide holder, wash the slides in 50mL in PBS for 5 minutes each, twice.
   b. Retrieve the IHC stains from the fridge in the histology lab, and prepare the stain using manufacturer's instructions.
   c. Add the IHC stain solution to the sections, vacuuming off the PBS solution from the section. This can be done over paper towel to allow for better visualization of the stain.
   d. Leave the IHC stain for 3 minutes, if needed, use visuals to determine when the sample is sufficiently stained.
   e. Rinse stains off using distilled water.
5. Mount (See Section 1.10)

## 1.5.2 Procedure for Mounting Slides

1. Dehydrate slides in 2 changes of 95% alcohol, and 2 changes of 100% ethanol for 2 minutes each.
2. Clear in 2 changes of xylene for 2 minutes each.

Place a small drop of mounting media on slide, place glass coverslip and using plastic tweezers compress and remove all air bubbles.

## 1.6 Summary of Differential Expression Results

Differential expression provided details of the raw, unadjusted p-value which provided details as to the up- and downregulation of good responders with respect to poor responders. Notably, the genes with the highest log2(Fold Change) were tryptase alpha/beta 1 (*TPSAB1*, 3.29), a mast cell-associated serine protease responsible for matric organization and defense response, the membrane metalloendopeptidase (*MME*, 2.75), a membrane-bound metallopeptidase, and tumor necrosis factor superfamily member 11B (*TNFRSF11B*, 2.61), which encodes OPG and is a regulator of bone remodeling. Other genes with a nominal p-value < 0.05 included fractalkine (*CX3CL1*, 1.93), a chemokine involved in leukocyte adhesion and migration, interleukin 11 receptor subunit alpha (*IL11RA*, 2.01), and interleukin 18 receptor 1 (*IL18R1*, 1.8). Additionally, protein kinase C epsilon (*PRKCE*, 1.45), a protein kinase involved in intracellular signalling, RAR-related orphan receptor alpha (*RORA*, 1.36), a transcription factor involved in cellular differentiation and immune regulation, and platelet-derived growth factor receptor beta (*PDGFRB*, 1.2), which is linked to angiogenesis and cell proliferation, had high log2(Fold Change).

Genes with the lowest log2(Fold change) were toll like receptor 10 (*TLR10*, −2.37), a gene involved in innate immune recognition and signalling, and CX3C motif chemokine receptor 1 (*CX3CR1*, −1.76), which is the receptor of fractalkine (upregulated in good responders), were significantly differentially expressed based on nominal p-values. T-box transcription factor 21 (*TBX21*, T-bet, −1.39), a regulator of Th1 cells, the CD40 ligand (*CD40LG*, −1.91), a gene critical in the activation of T- and B-cells, and arginase 2 (*ARG2*, −1.47), which is linked to arginine metabolism in macrophages, were also downregulated based on nominal p-values < 0.05. Other downregulated genes included the T-cell immunoreceptor with Ig and ITIM domains (*TIGIT*, −1.37), associated with immune regulation involving T-cells and natural killer cells, and CXC motif chemokine ligand 1 *(CXCL1*, −1.77), a chemokine that promotes inflammation, neutrophil chemotaxis, and has also been associated with tumor growth and progression.

Of the genes with nominal p-values < 0.05, *CX3CL1, TPSAB1*, and *TNFRSF11B* had the lowest nominal p-values (0.000168, 0.000403, and 0.000415, respectively) and Benjamini–Yekutieli adjusted p-values of 0.46. All other genes had Benjamini–Yekutieli adjusted p-values of 1.00. Thus, none of the genes met a Benjamini–Yekutieli adjusted p-value threshold of 0.05, nevertheless, these genes remain of interest based on their observed log_2_(Fold Change) and nominal p-values.

# 2 Supplemental Figures and Analysis

## 2.1 Classifications of Ossification Based on Boriani Grading by Surgeon

The surgeons graded tumor ossification using clinical CT imaging in 3-month increments. The tumors were graded based on the previously defined Boriani Grade^1^ further depicted in Figure S1.

Figure S1. The classification system grades a tumor between 1 and 6, corresponding to the six different Boriani Grades. A grade of 0 indicates no visible ossification within the tumor, or on the peripheral edges. A grade 1 indicates no internal ossification but is split into two sub-grades based on the amount of peripheral rim (a, incomplete; b, complete). A grade 2 indicates some internal ossification, and an incomplete peripheral rim (a, scattered and sporadic ossification; b, 50% internal ossification). A grade 3 indicates high internal ossification at greater than 50%, and a complete peripheral rim.

## 2.2 Unsupervised clustering shows minimal grouping based on response type

In the initial analysis, which includes 773 genes detected, we observed no clear clustering of good responders and poor responders. This analysis was completed with unknown responders (patients that did not have ossification related Boriani grading on file) included. After unsupervised clustering, regardless of minimal clustering, the two unknowns were grouped based on their independent clustering to allow for their inclusion in differential expression analysis. For example, Sample 14 has clustered with Sample 7, a poor responder, and Sample 19’s nearest neighbouring clusters are good responders (Figure S2, outlined in red).

Figure S2. Visualization of gene clustering. A normalized heatmap identifies four clusters, with poor responders and good responders primarily grouped. Samples 2, 9, 16, and 17 show exceptions to this grouping. Samples 14 and 19 had unknown response types due to incomplete tumor ossification grading and were regrouped based on their clustering (outlined in red).

## 2.3 Sensitivity analysis identifies high correlation between results of differential expression on all 19 samples when compared to results if samples with unknown Boriani classifications are excluded.

Figure S4. A) Volcano plot illustrating results of DE with baseline as bad, when samples with unknown Boriani grade are excluded. B) Volcano plot illustrating results of DE with baseline as bad, when samples with unknown Boriani grade are reclassified based on unsupervised clustering. C) Genes included in the correlation plot met the predefined differential expression criteria (|log₂FC| > 1 and p < 0.05) and appeared in both datasets (19 samples, 17 samples with unknowns excluded). From the 51 and 35 genes meeting the differential expression criteria in the 17- and 19-sample analyses, 23 were shared.

## 2.4 Histological and radiographic ossification is observed during denosumab treatment in cases of giant cell tumors of the spine.

 Histological analysis was performed on initial biopsied tissues (Figure S2.i). Hematoxylin and eosin staining is completed to diagnose giant cell tumors of bone (GCTB) and identify the regions with giant cells. These regions containing giant cells were used in the nanoString gene expression analysis. Post treatment staining shows the elimination of giant cells, and formation of woven bone. Additional radiographic imaging (Figure S2.ii, S2.A.iii)., high resolution microCT (Figure S2.Biii), and scanning electron microscopy (Figure S2.B.iv) were then used to identify qualitative and quantitative amounts of ossification of the tumors.

Figure S3. Before and after treatment ossification results of poor responder, Sample X. A) Pre-treatment imaging, i) H&E stained histological section showing groups of giant cells, ii) axial view from clinical CT shows a soft tissue void in the ventral body (closed arrows), extending to the spinous process, iii) a sagittal view from clinical MRI shows the soft tissue void remaining within the excised tumor (closed arrows). B) Post-treatment imaging, i) H&E stained histological section showing both lamellar and woven trabecular bone formation, ii) axial view from clinical CT shows scattered islands of ossification (open arrows), and an incomplete peripheral rim, iii) a microCT resliced axial cross section of the tumor, showing the scattered islands (open arrows) at a higher resolution (10μm isotropic voxel size), and iv) a stitched SEM image at 50x demonstrating low density bone formation within the tumoral space (open arrows).

## 2.5 Pathway and cell scores demonstrate differences based on response type, providing insight into the tumor microenvironment

Figure S5. Pathway scores for pathway versus response type for A) cell cycle, B) chemokine, C) cytokine, D) cytotoxicity, E) interleukins, F) T-cell functions, and G) regulation pathways.

Figure S6. Cell scores for cell types versus response type for A) cytotoxic cells, B) dendritic cells (DC), C) macrophages, D) mast cells, E) neutrophils, F) natural killer (CD56dim) cells, G) T-cells, and H) Th1 cells.

## 2.6 Proteomic profiles suggest differences in giant cell tumor biopsies from healthy control vertebral cores

To confirm the genetic differences observed and a patients’ predisposition to denosumab treatment, we followed our study with proteomics analysis of 6 pre-treatment biopsies. Across two combined runs, a total of 8,307 proteins were identified. Principal component analysis (PCA, Fig. S6) was first performed to assess overall variation in the proteomics dataset, followed by unsupervised clustering of the GCTB biopsy and control samples (Figure S6A). The control samples clustered separately from the GCTB biopsy samples, with the exception of one good responder (Sample 10), which clustered with the controls consistently before and after imputation (Figure S7). The raw expression values were then used for exploratory comparison to identify if the expression was higher or lower than that observed in normal bone (Figure S7B). While patterns of expression could be identified between the GCTB samples based on response classification, minimal trends could be observed with respect to the healthy bone samples.

Figure S7. PCA analysis showing distinct clustering of healthy controls from GCTB samples, A) before imputation, and B) after imputation. Missingness rate for the GCTB samples only can be found in Table S8.

Figure S8. Visualization of protein clustering and expression levels. A) A normalized heatmap post-imputation identifies two main clusters, with controls and GCTB samples primarily grouped. Sample 10 has clustered with the control samples, and Sample 6 (good responder) clusters with the poor responders. B) An illustration of the protein expression levels after normalization and imputation, mapped to its respective gene name.

## 2.9 Immunohistochemistry demonstrates spatial localization of exploratory markers, with broad mineralization indicated by osteocalcin and low osteoblastic activity in poor responders.

## Figure S10. Immunohistochemistry of exploratory proteins to identify protein localization and bone formation activity in GCTB. Scale = 1 mm, magnification = 2.5X.

## 2.10 Differences in tumor microenvironment contribute to increased mineralization outcomes in good responders compared to poor responders.

We have previously reported varying amounts of woven bone formed during denosumab treatment^2^. We used previously reported microCT data and mineral content measured from qBSE from 4 of 19 patients who had their tumors, surgically removed, post-denosumab treatment, to determine if observations may suggest a possible relationship between the biomarkers identified in the pre-treatment biopsies, and the mineral content of the lesions post treatment measured as mineral content and BV/TV. In our small cohort with recorded mineral data (n=4), mineral content has a strong positive trend with CX3CR1 and a very strong positive trend with CX3CL1 (0.63 and 0.80, respectively) but these relationships should be interpreted cautiously given the small sample size and were weakly related to BV/TV. The two mineralization outcomes show a weak positive trend consistent with higher TNFRSF11B expression (Figure 5, 0.40 and 0.20), respectively determined using Spearman correlation). Larger changes in the tumor Boriani grade shows a strong tendency to increase with CX3CL1 (0.74). While we had additional protein expression data, only two samples had post-treatment mineral data collected and was therefore excluded from this correlative analysis. Moreover, this small cohort only demonstrates Additionally, this small cohort demonstrates exploratory correlations that should not be interpreted as predictive or mechanistic relationships, and the limitations of this small sample size must be considered. These findings do not establish a simple high- or low-expression relationship between CX3CL1/CX3CR1 and subsequent mineralization. Future studies should explore the links between pre-treatment tumor characteristics and the post-treatment mineralization characteristics in a larger cohort. Our recommendation is that future studies should explore the links between pre-treatment tumor characteristics and the post-treatment mineralization characteristics in a larger cohort.

Figure S9. Spearman correlation analysis demonstrates positive association between mineralization parameters (BV/TV and mineral content) with raw gene expression counts in 4 of 19 patients who underwent post-denosumab resection. Mineral content and BV/TV have been previously summarized ^2^.

3 Supplemental Tables

Table S1. A summary of antibodies used for independent validation of candidate markers.

| Antibody Name | Supplier | Lot/Batch Number | Concentration |
| --- | --- | --- | --- |
| Rabbit monoclonal Collagen I (COL1) | Abcam |  | 1:100 |
| Rabbit polyclonal Runt-related transcription factor 2 (RUNX2) | Protein-Tech | 82636-2-RR | 1:200 |
| Rabbit polyclonal tartrate-resistant acid phosphatase (TRAP) | Abcam |  | 1:100 |
| Rabbit polyclonal Osteocalcin (OCN) | Abcam | GR3439937-2 | 1:100 |
| Rabbit polyclonal Osteoprotegerin (OPG) | Abcam | AB 73400 | 1:1000 |
| Rabbit polyclonal Fractalkine (CX3CL1) | Abcam | AB 25088 | 1:1000 |
| Rabbit monoclonal Cathepsin K (CTSK) | Abcam | AB 1876647 | 1:1000 |

Table S2. A summary of pre-denosumab treated patient demographics, used in biopsies and genetic study.

| ID | Sex | Age  (yrs.) | Treatment Duration (months) | Responder Type | Boriani Classification | | | Metastatic | Deceased |
| --- | --- | --- | --- | --- | --- | --- | --- | --- | --- |
|  |  |  |  |  | *Pre-Dmab* | *At 6 months* | *Post-Dmab* |  |  |
| 1 | F | 57 | Unknown | Good | 2 | 5 | 5 | Unknown | No |
| 2 | F | 59 | 6 | Poor | 1 | 2 | 2 | Yes | Yes |
| 3 | F | 45 | 6 | Good | 2 | 4 | 5 | Unknown | Unknown |
| 4 | M | 40 | 6.5 | Poor | 1 | 2 | 2 | Yes | Yes |
| 5 | M | 30 | 5 | Good | 2 | 6 | 6 | Unknown | Unknown |
| 6 | M | 63 | 8 | Good | 1 | 4 | 4 | Unknown | Unknown |
| 7 | F | 55 | 16 | Poor | 2 | 2 | 2 | No | No |
| 8 | M | 24 | 3 | Poor | 4 | 5 | 5 | No | No |
| 9 | F | 20 | Unknown | Good | 2 | 6 | 6 | No | No |
| 10 | F | 21 | 8 | Good | 4 | 6 | 6 | Unknown | No |
| 11 | F | 23 | 10 | Poor | 3 | 3 | 3 | Yes | No |
| 12 | F | 23 | Ongoing | Poor | 3 | 3 | 3 | No | No |
| 13 | F | 15 | Ongoing | Poor | 3 | 3 | 3 | No | No |
| 14 | M | 18 | 11 | Unknown | Unknown | Unknown | Unknown | No | No |
| 15 | F | 23 | 25 | Good | 2 | 5 | 5 | No | No |
| 16 | M | 23 | 11 | Poor | 1 | 2 | 4 | No | No |
| 17 | M | 52 | 4 | Poor | 4 | 4 | 4 | No | No |
| 18 | M | 41 | Unknown | Good | 1 | 4 | 4 | No | No |
| 19 | M | 24 | 27.5 | Unknown | Unknown | Unknown | Unknown | Unknown | No |

Table S3. A summary of healthy control patient demographics.

| ID | Sex | Age (yrs.) | Cause of Death | History of Bone Related Disease |
| --- | --- | --- | --- | --- |
| 1 | M | 87 | Natural Causes | No |
| 2 | M | 81 | Heart Attack | No |
| 3 | M | 52 | MAiD | No |

Table S4. A summary of normalization factors used in gene expression positive control normalization.

| ID | *Batch* | *mRNA Positive Normalization Factor* | *mRNA Content Normalization Factor* |
| --- | --- | --- | --- |
| 1 | 2 | 1.0009503 | 0.69835865 |
| 1 | 1 | 1.0745199 | 1.0691546 |
| 2 | 1 | 0.89598656 | 1.5838361 |
| 3 | 1 | 0.817827 | 2.3970687 |
| 4 | 1 | 0.8548589 | 10.142838 |
| 5 | 2 | 0.96758276 | 1.0412879 |
| 5 | 1 | 0.799056 | 2.056416 |
| 6 | 1 | 0.9448918 | 1.2370186 |
| 7 | 1 | 1.1119449 | 1.3251687 |
| 8 | 2 | 1.0964395 | 4.2019577 |
| 9 | 2 | 2.1609926 | 0.2328126 |
| 10 | 2 | 0.98383427 | 1.4619508 |
| 11 | 1 | 0.80436724 | 8.708185 |
| 12 | 2 | 1.9314922 | 0.50525194 |
| 13 | 1 | 0.8884874 | 2.5673583 |
| 14 | 2 | 1.1241254 | 0.44532862 |
| 15 | 2 | 1.1633805 | 0.7150142 |
| 16 | 2 | 0.96886814 | 0.6553673 |
| 17 | 2 | 0.8518364 | 1.5489655 |
| 18 | 2 | 0.82890886 | 1.1409909 |
| 19 | 2 | 1.5044111 | 1.0949069 |
| Control | 1 | 0.8230169 | 5.8894362 |

Table S5. BCA protein concentrations.

| ID | *Protein BCA*  *[μg/μL]* | *Peptide BCA pre-SPE [μg/μL]* | *Peptide BCA post-SPE [μg/μL]* | *Sample Volume [μL]* | *0.1% TFA Volume [μL]* |
| --- | --- | --- | --- | --- | --- |
| 6-A | 1.461 | 1.58 | 2.79 | 1.4 | 6.6 |
| 6-B | 1.299 | 1.69 | 2.90 | 1.4 | 6.6 |
| 7-A | 0.256 | 0.24 | 0.57 | 7.0 | 1.0 |
| 7-B | 0.225 | 0.56 | 1.14 | 3.5 | 4.5 |
| 8 | 0.858 | 3.45 | 5.11 | 0.8 | 7.2 |
| 9 | 0.966 | 1.15 | 1.72 | 2.3 | 5.7 |
| 10 | 0.416 | 1.51 | 1.22 | 3.3 | 4.7 |
| 12 | 1.059 | 1.89 | 2.05 | 1.9 | 6.1 |

Table S6. Pathways from Proteomic Sensitivity Analysis that Showed Disagreement

| Pathway | *NES (0.75 Missingness Threshold)* | *NES (0.5 Missingness Threshold)* | *NES (0.25 Missingness Threshold)* |
| --- | --- | --- | --- |
| Osteoblast Differentiation | 0.9638224 | 0.9329866 | N/A |
| Regulation of Bone Remodeling | -1.3782477 | N/A | -1.3969563 |
| Skeletal Muscle Cell Differentiation | N/A | -1.0668669 | -1.1366293 |

Table S7. Raw and adjusted p-values from differential expression. Both the Benjamini-Yekutieli (BY) and Benjamini-Hochberg (BH) adjusted p-values are reported to reflect that the BY method, which is more conservative, shows slightly less significance than the BH method.

| Gene Name | Raw p-value | BY adjusted p-value | BH adjusted p-value |
| --- | --- | --- | --- |
| CX3CL1 | *0.000168* | *0.46* | *0.0679* |
| TPSAB1 | *0.000403* | *0.46* | *0.0679* |
| TNFRSF11B | *0.000415* | *0.46* | *0.0679* |
| PRKCE | *0.00311* | *1* | *0.19* |
| MME | *0.00352* | *1* | *0.19* |
| TLR10 | *0.00387* | *1* | *0.19* |
| CX3CR1 | *0.00404* | *1* | *0.19* |
| IL11RA | *0.00427* | *1* | *0.19* |
| CD200 | *0.00497* | *1* | *0.191* |
| IL18R1 | *0.00751* | *1* | *0.263* |
| CD40LG | *0.00918* | *1* | *0.282* |
| TAL1 | *0.0136* | *1* | *0.316* |
| C5 | *0.0137* | *1* | *0.316* |
| TNFRSF4 | *0.0148* | *1* | *0.316* |
| ARG2 | *0.0163* | *1* | *0.334* |
| TIGIT | *0.0204* | *1* | *0.385* |
| CXCL1 | *0.0245* | *1* | *0.412* |
| CR2 | *0.0265* | *1* | *0.412* |
| TBX21 | *0.0268* | *1* | *0.412* |
| NOD1 | *0.0292* | *1* | *0.412* |
| CCL20 | *0.0301* | *1* | *0.412* |
| CMKLR1 | *0.0397* | *1* | *0.412* |
| CD34 | *0.0426* | *1* | *0.412* |

Table S8. Differential expression results for all genes displayed in the volcano plot in Figure 2 of the main text.

| Gene ID | Log_2_(FC) | Std Error (log_2_) | Lower confidence limit (log_2_) | Upper confidence limit (log_2_) | P-value | BY  P-value |
| --- | --- | --- | --- | --- | --- | --- |
| CX3CL1 | 1.93 | 0.38 | 1.18 | 2.67 | 0.000168 | 0.46 |
| TPSAB1 | 3.29 | 0.713 | 1.89 | 4.68 | 0.000403 | 0.46 |
| TNFRSF11B | 2.61 | 0.568 | 1.5 | 3.72 | 0.000415 | 0.46 |
| AKT3 | 0.854 | 0.208 | 0.448 | 1.26 | 0.00105 | 0.871 |
| PDGFRB | 1.2 | 0.316 | 0.582 | 1.82 | 0.00194 | 1 |
| RORA | 1.36 | 0.377 | 0.622 | 2.1 | 0.00285 | 1 |
| PRKCE | 1.45 | 0.407 | 0.653 | 2.25 | 0.00311 | 1 |
| MME | 2.75 | 0.786 | 1.21 | 4.29 | 0.00352 | 1 |
| TLR10 | -2.37 | 0.685 | -3.71 | -1.02 | 0.00387 | 1 |
| CX3CR1 | -1.76 | 0.512 | -2.76 | -0.754 | 0.00404 | 1 |
| IL11RA | 2.01 | 0.59 | 0.853 | 3.16 | 0.00427 | 1 |
| CD200 | 1.03 | 0.309 | 0.423 | 1.64 | 0.00497 | 1 |
| ABL1 | 0.693 | 0.209 | 0.284 | 1.1 | 0.00507 | 1 |
| IL18R1 | 1.8 | 0.576 | 0.669 | 2.93 | 0.00751 | 1 |
| MR1 | -0.885 | 0.289 | -1.45 | -0.317 | 0.00855 | 1 |
| CD40LG | -1.91 | 0.632 | -3.15 | -0.67 | 0.00918 | 1 |
| TNFRSF18 | 1.5 | 0.52 | 0.483 | 2.52 | 0.0119 | 1 |
| IRF2 | -0.827 | 0.293 | -1.4 | -0.253 | 0.0136 | 1 |
| CCRL2 | -0.78 | 0.276 | -1.32 | -0.238 | 0.0136 | 1 |
| TAL1 | 1.39 | 0.493 | 0.423 | 2.35 | 0.0136 | 1 |
| C5 | 1.66 | 0.588 | 0.505 | 2.81 | 0.0137 | 1 |
| BATF | -1.02 | 0.364 | -1.73 | -0.305 | 0.0142 | 1 |
| TNFRSF4 | 1.1 | 0.396 | 0.324 | 1.88 | 0.0148 | 1 |
| ARG2 | -1.47 | 0.537 | -2.52 | -0.413 | 0.0163 | 1 |
| TP53 | 0.396 | 0.15 | 0.102 | 0.69 | 0.0193 | 1 |
| TIGIT | -1.37 | 0.526 | -2.4 | -0.344 | 0.0204 | 1 |
| IL6ST | 0.824 | 0.323 | 0.192 | 1.46 | 0.0229 | 1 |
| CXCL1 | -1.77 | 0.704 | -3.15 | -0.394 | 0.0245 | 1 |
| CR2 | -1.5 | 0.606 | -2.69 | -0.315 | 0.0265 | 1 |
| GZMB | -2.19 | 0.885 | -3.92 | -0.455 | 0.0268 | 1 |
| TBX21 | -1.39 | 0.561 | -2.49 | -0.288 | 0.0268 | 1 |
| NOD1 | 1.11 | 0.458 | 0.215 | 2.01 | 0.0292 | 1 |
| CCL20 | -1.45 | 0.599 | -2.62 | -0.272 | 0.0301 | 1 |
| CD99 | 1 | 0.418 | 0.183 | 1.82 | 0.031 | 1 |
| CYFIP2 | -1.13 | 0.476 | -2.06 | -0.197 | 0.0325 | 1 |
| VEGFA | -1.55 | 0.656 | -2.84 | -0.266 | 0.033 | 1 |
| SAA1 | -1.7 | 0.732 | -3.14 | -0.267 | 0.0356 | 1 |
| STAT2 | 0.966 | 0.416 | 0.151 | 1.78 | 0.0358 | 1 |
| IL12RB1 | -1.07 | 0.468 | -1.99 | -0.155 | 0.0379 | 1 |
| CMKLR1 | 1.23 | 0.543 | 0.167 | 2.29 | 0.0397 | 1 |
| ZAP70 | -1.26 | 0.559 | -2.35 | -0.162 | 0.041 | 1 |
| CD34 | 2.02 | 0.907 | 0.245 | 3.8 | 0.0426 | 1 |
| CD276 | 0.745 | 0.336 | 0.0852 | 1.4 | 0.044 | 1 |
| ATG10 | 2.19 | 0.998 | 0.234 | 4.15 | 0.0455 | 1 |
| ATG16L1 | 1.83 | 0.838 | 0.192 | 3.48 | 0.046 | 1 |
| CREB1 | 0.65 | 0.298 | 0.0661 | 1.23 | 0.0467 | 1 |
| STAT3 | 0.62 | 0.287 | 0.057 | 1.18 | 0.0488 | 1 |
| TAB1 | 2.27 | 1.07 | 0.174 | 4.36 | 0.0521 | 1 |
| LCK | -1.01 | 0.476 | -1.94 | -0.0744 | 0.0527 | 1 |
| SOCS1 | 1.38 | 0.652 | 0.101 | 2.66 | 0.0528 | 1 |
| TIRAP | 1.88 | 0.89 | 0.132 | 3.62 | 0.0535 | 1 |
| EWSR1 | 0.695 | 0.33 | 0.0477 | 1.34 | 0.0539 | 1 |
| PPARG | 1.44 | 0.684 | 0.0958 | 2.78 | 0.0543 | 1 |
| FAS | 1.12 | 0.534 | 0.0685 | 2.16 | 0.0555 | 1 |
| STAT5B | 2.21 | 1.06 | 0.134 | 4.29 | 0.0557 | 1 |
| BMI1 | 2.28 | 1.09 | 0.134 | 4.42 | 0.0561 | 1 |
| FLT3LG | 1.25 | 0.6 | 0.0721 | 2.42 | 0.0564 | 1 |
| FYN | 0.733 | 0.353 | 0.0409 | 1.42 | 0.0568 | 1 |
| YTHDF2 | 2.46 | 1.19 | 0.133 | 4.78 | 0.0572 | 1 |
| IL4R | 2.7 | 1.31 | 0.144 | 5.26 | 0.0574 | 1 |
| IRAK2 | 1.14 | 0.552 | 0.0522 | 2.22 | 0.0591 | 1 |
| MAPK3 | 2.47 | 1.21 | 0.112 | 4.84 | 0.0593 | 1 |
| ILF3 | 0.577 | 0.282 | 0.0234 | 1.13 | 0.0604 | 1 |
| RELB | 2.13 | 1.06 | 0.0624 | 4.2 | 0.0631 | 1 |
| CXCL9 | 1.41 | 0.698 | 0.0393 | 2.77 | 0.0634 | 1 |
| TAP1 | 2.17 | 1.08 | 0.059 | 4.29 | 0.0636 | 1 |
| ABCB1 | 1.23 | 0.616 | 0.027 | 2.44 | 0.0648 | 1 |
| PNMA1 | 2.4 | 1.2 | 0.0517 | 4.75 | 0.0649 | 1 |
| IL1B | -1.88 | 0.941 | -3.73 | -0.0377 | 0.0653 | 1 |
| IL17RA | -0.539 | 0.27 | -1.07 | -0.0099 | 0.0657 | 1 |
| IFIT2 | 1.48 | 0.742 | 0.0219 | 2.93 | 0.0665 | 1 |
| IRAK4 | 2.07 | 1.04 | 0.0255 | 4.12 | 0.0672 | 1 |
| ETS1 | 0.718 | 0.362 | 0.00843 | 1.43 | 0.0673 | 1 |
| TNFSF12 | 2.52 | 1.27 | 0.0292 | 5.02 | 0.0673 | 1 |
| TRAF6 | 2.29 | 1.15 | 0.0262 | 4.55 | 0.0674 | 1 |
| CCND3 | 2.41 | 1.22 | 0.0252 | 4.8 | 0.0676 | 1 |
| IL10 | -1.17 | 0.593 | -2.34 | -0.0092 | 0.0683 | 1 |
| COLEC12 | 0.728 | 0.369 | 0.0055 | 1.45 | 0.0683 | 1 |
| SMAD2 | 2.34 | 1.19 | 0.0148 | 4.67 | 0.0686 | 1 |
| NOD2 | -1.3 | 0.662 | -2.6 | -0.00653 | 0.069 | 1 |
| MAVS | 2.68 | 1.36 | 0.00508 | 5.35 | 0.0697 | 1 |
| PIN1 | 1.63 | 0.831 | -0.00252 | 3.25 | 0.0706 | 1 |
| MAP2K4 | 2.05 | 1.05 | -0.00427 | 4.11 | 0.0707 | 1 |
| IL15 | -0.485 | 0.249 | -0.972 | 0.0028 | 0.0717 | 1 |
| CDH5 | 1.08 | 0.557 | -0.00747 | 2.17 | 0.0719 | 1 |
| TBK1 | 0.377 | 0.194 | -0.00286 | 0.756 | 0.0721 | 1 |
| PSEN1 | 2.43 | 1.25 | -0.0285 | 4.88 | 0.0732 | 1 |
| IFI16 | 2.66 | 1.38 | -0.05 | 5.38 | 0.0749 | 1 |
| TNFSF4 | -1.08 | 0.57 | -2.2 | 0.0325 | 0.0778 | 1 |
| MAPK8 | 1.39 | 0.732 | -0.0419 | 2.83 | 0.0778 | 1 |
| RIPK2 | 2.11 | 1.11 | -0.0727 | 4.3 | 0.079 | 1 |
| SMAD3 | 2.09 | 1.11 | -0.0803 | 4.26 | 0.08 | 1 |
| TRAF2 | 0.451 | 0.239 | -0.0181 | 0.92 | 0.0804 | 1 |
| RELA | 1.94 | 1.03 | -0.0795 | 3.96 | 0.0807 | 1 |
| CHUK | 2.19 | 1.17 | -0.106 | 4.48 | 0.0826 | 1 |
| MAP2K1 | -0.255 | 0.139 | -0.528 | 0.0169 | 0.0873 | 1 |
| TICAM1 | 2.01 | 1.09 | -0.134 | 4.15 | 0.0875 | 1 |
| NCF4 | 2.48 | 1.35 | -0.17 | 5.12 | 0.088 | 1 |
| DDX58 | 1.01 | 0.554 | -0.0749 | 2.1 | 0.0894 | 1 |
| TREM1 | -1.56 | 0.858 | -3.24 | 0.121 | 0.0904 | 1 |
| TRAF3 | 2.18 | 1.2 | -0.173 | 4.53 | 0.0909 | 1 |
| CCL17 | -1.28 | 0.706 | -2.66 | 0.107 | 0.092 | 1 |
| TNFRSF1A | 0.519 | 0.287 | -0.0433 | 1.08 | 0.092 | 1 |
| MICA | 1.79 | 0.99 | -0.152 | 3.73 | 0.0924 | 1 |
| RUNX3 | 2.22 | 1.23 | -0.198 | 4.63 | 0.0935 | 1 |
| CXCL3 | -1.29 | 0.721 | -2.71 | 0.119 | 0.0943 | 1 |
| HLA-G | -0.698 | 0.391 | -1.46 | 0.0679 | 0.0957 | 1 |
| CSF1R | 2.23 | 1.25 | -0.22 | 4.67 | 0.0962 | 1 |
| CD33 | 2.02 | 1.14 | -0.219 | 4.27 | 0.0988 | 1 |
| TNFRSF11A | 2.56 | 1.45 | -0.282 | 5.4 | 0.0992 | 1 |
| CREB5 | 0.392 | 0.224 | -0.0471 | 0.831 | 0.102 | 1 |
| TICAM2 | 2.06 | 1.18 | -0.248 | 4.38 | 0.102 | 1 |
| LRP1 | 0.596 | 0.344 | -0.0772 | 1.27 | 0.105 | 1 |
| CXCL2 | -1.26 | 0.732 | -2.69 | 0.176 | 0.107 | 1 |
| HLA-DOB | -1.13 | 0.657 | -2.42 | 0.155 | 0.107 | 1 |
| MAPK14 | 0.333 | 0.194 | -0.0463 | 0.713 | 0.107 | 1 |
| TLR8 | -0.792 | 0.462 | -1.7 | 0.112 | 0.108 | 1 |
| ICAM1 | 2.78 | 1.62 | -0.397 | 5.95 | 0.108 | 1 |
| PIK3CG | 2.15 | 1.26 | -0.311 | 4.62 | 0.109 | 1 |
| CSF1 | 2.51 | 1.47 | -0.372 | 5.39 | 0.11 | 1 |
| LTBR | 0.512 | 0.301 | -0.0774 | 1.1 | 0.111 | 1 |
| ITGB3 | 2.79 | 1.64 | -0.423 | 6.01 | 0.111 | 1 |
| IL32 | 1.04 | 0.613 | -0.163 | 2.24 | 0.112 | 1 |
| SPN | 1.11 | 0.654 | -0.173 | 2.39 | 0.112 | 1 |
| JAK3 | 1.13 | 0.664 | -0.174 | 2.43 | 0.112 | 1 |
| CD97 | 0.308 | 0.183 | -0.0505 | 0.666 | 0.114 | 1 |
| LAMP2 | 0.607 | 0.36 | -0.0983 | 1.31 | 0.114 | 1 |
| ATF2 | 2.11 | 1.26 | -0.354 | 4.58 | 0.115 | 1 |
| IFI27 | 2.47 | 1.47 | -0.42 | 5.36 | 0.116 | 1 |
| COL3A1 | 1.2 | 0.723 | -0.213 | 2.62 | 0.118 | 1 |
| JAK1 | 1.8 | 1.09 | -0.334 | 3.94 | 0.12 | 1 |
| SLC11A1 | -1.15 | 0.697 | -2.52 | 0.214 | 0.121 | 1 |
| NCAM1 | 1.55 | 0.939 | -0.291 | 3.39 | 0.121 | 1 |
| HRAS | 1.49 | 0.906 | -0.283 | 3.27 | 0.122 | 1 |
| APP | 0.625 | 0.383 | -0.126 | 1.38 | 0.125 | 1 |
| LTK | 1.22 | 0.75 | -0.247 | 2.69 | 0.125 | 1 |
| IGF2R | 1.7 | 1.05 | -0.361 | 3.77 | 0.128 | 1 |
| CD63 | 0.54 | 0.339 | -0.124 | 1.2 | 0.133 | 1 |
| MAP2K2 | 0.651 | 0.409 | -0.152 | 1.45 | 0.134 | 1 |
| SPA17 | 0.912 | 0.574 | -0.214 | 2.04 | 0.135 | 1 |
| CCL3 | -0.653 | 0.419 | -1.47 | 0.168 | 0.141 | 1 |
| IGF1R | 0.444 | 0.287 | -0.118 | 1.01 | 0.144 | 1 |
| TFE3 | 0.402 | 0.262 | -0.111 | 0.915 | 0.146 | 1 |
| IRF1 | 0.727 | 0.474 | -0.202 | 1.66 | 0.147 | 1 |
| MAF | 1.12 | 0.731 | -0.31 | 2.56 | 0.147 | 1 |
| EP300 | 0.509 | 0.333 | -0.143 | 1.16 | 0.148 | 1 |
| C1S | 0.592 | 0.388 | -0.167 | 1.35 | 0.149 | 1 |
| ICAM2 | 0.833 | 0.551 | -0.247 | 1.91 | 0.153 | 1 |
| ATG12 | 0.7 | 0.465 | -0.21 | 1.61 | 0.154 | 1 |
| JAM3 | 0.818 | 0.543 | -0.246 | 1.88 | 0.154 | 1 |
| CD55 | 0.685 | 0.456 | -0.207 | 1.58 | 0.155 | 1 |
| ECSIT | 0.624 | 0.416 | -0.192 | 1.44 | 0.156 | 1 |
| PSMD7 | 1.26 | 0.841 | -0.387 | 2.91 | 0.156 | 1 |
| NFATC4 | 1.26 | 0.843 | -0.391 | 2.91 | 0.157 | 1 |
| VEGFC | -0.986 | 0.662 | -2.28 | 0.312 | 0.159 | 1 |
| PLAUR | -0.719 | 0.49 | -1.68 | 0.241 | 0.164 | 1 |
| LY96 | 1.94 | 1.33 | -0.665 | 4.55 | 0.166 | 1 |
| LAMP1 | 0.504 | 0.347 | -0.177 | 1.18 | 0.169 | 1 |
| PPBP | 1.26 | 0.873 | -0.451 | 2.97 | 0.171 | 1 |
| MAP3K7 | 0.309 | 0.215 | -0.112 | 0.73 | 0.172 | 1 |
| REPS1 | 1.27 | 0.884 | -0.463 | 3 | 0.173 | 1 |
| A2M | 0.954 | 0.666 | -0.351 | 2.26 | 0.174 | 1 |
| BTK | -0.347 | 0.245 | -0.827 | 0.134 | 0.179 | 1 |
| TTK | 0.837 | 0.592 | -0.323 | 2 | 0.179 | 1 |
| ATG5 | -0.269 | 0.192 | -0.645 | 0.107 | 0.182 | 1 |
| IFIH1 | 1.48 | 1.06 | -0.591 | 3.55 | 0.183 | 1 |
| UBC | 0.45 | 0.324 | -0.184 | 1.08 | 0.186 | 1 |
| CD3EAP | 0.466 | 0.335 | -0.191 | 1.12 | 0.186 | 1 |
| NLRC5 | 0.812 | 0.584 | -0.332 | 1.96 | 0.186 | 1 |
| CD84 | 1.93 | 1.39 | -0.796 | 4.66 | 0.187 | 1 |
| CEBPB | -0.607 | 0.439 | -1.47 | 0.253 | 0.188 | 1 |
| STAT6 | 0.28 | 0.203 | -0.118 | 0.678 | 0.189 | 1 |
| PECAM1 | 0.584 | 0.423 | -0.246 | 1.41 | 0.189 | 1 |
| BCL2 | 0.686 | 0.497 | -0.289 | 1.66 | 0.189 | 1 |
| CD24 | -1.27 | 0.93 | -3.09 | 0.553 | 0.194 | 1 |
| C1QBP | 1.11 | 0.813 | -0.484 | 2.7 | 0.194 | 1 |
| CREBBP | -0.502 | 0.37 | -1.23 | 0.223 | 0.196 | 1 |
| CD47 | 0.484 | 0.36 | -0.221 | 1.19 | 0.2 | 1 |
| CASP8 | 1.26 | 0.936 | -0.579 | 3.09 | 0.201 | 1 |
| CCL18 | 2.18 | 1.63 | -1.01 | 5.38 | 0.202 | 1 |
| TNFRSF1B | -0.61 | 0.456 | -1.5 | 0.285 | 0.203 | 1 |
| ITGAE | -0.406 | 0.305 | -1 | 0.192 | 0.204 | 1 |
| CD7 | -0.803 | 0.604 | -1.99 | 0.38 | 0.205 | 1 |
| OAS3 | 0.75 | 0.564 | -0.356 | 1.86 | 0.205 | 1 |
| CD46 | 1.56 | 1.18 | -0.743 | 3.87 | 0.205 | 1 |
| CXCL12 | 1.07 | 0.813 | -0.526 | 2.66 | 0.21 | 1 |
| NFATC1 | 0.507 | 0.387 | -0.253 | 1.27 | 0.212 | 1 |
| IL8 | -2.16 | 1.66 | -5.42 | 1.09 | 0.214 | 1 |
| JAK2 | 0.381 | 0.292 | -0.192 | 0.954 | 0.214 | 1 |
| ITGA1 | 1.8 | 1.38 | -0.912 | 4.52 | 0.214 | 1 |
| TLR3 | 0.524 | 0.405 | -0.269 | 1.32 | 0.216 | 1 |
| MAPKAPK2 | 0.343 | 0.265 | -0.176 | 0.862 | 0.217 | 1 |
| CCL14 | 0.575 | 0.447 | -0.301 | 1.45 | 0.219 | 1 |
| NOTCH1 | 1.22 | 0.953 | -0.647 | 3.09 | 0.221 | 1 |
| ITCH | 1.41 | 1.1 | -0.748 | 3.57 | 0.221 | 1 |
| C4B | -0.809 | 0.633 | -2.05 | 0.431 | 0.222 | 1 |
| IL13RA1 | 0.329 | 0.26 | -0.181 | 0.839 | 0.227 | 1 |
| CD164 | 0.566 | 0.45 | -0.316 | 1.45 | 0.229 | 1 |
| KLRC2 | -1.02 | 0.813 | -2.61 | 0.576 | 0.231 | 1 |
| TOLLIP | 0.428 | 0.343 | -0.243 | 1.1 | 0.232 | 1 |
| IKBKG | 1.12 | 0.898 | -0.64 | 2.88 | 0.233 | 1 |
| CLEC5A | -1 | 0.807 | -2.59 | 0.578 | 0.234 | 1 |
| MAPK11 | 0.711 | 0.572 | -0.41 | 1.83 | 0.234 | 1 |
| PSEN2 | 0.738 | 0.597 | -0.432 | 1.91 | 0.237 | 1 |
| IL2RB | 0.762 | 0.622 | -0.457 | 1.98 | 0.241 | 1 |
| CDK1 | 0.85 | 0.697 | -0.515 | 2.22 | 0.242 | 1 |
| BCL2L1 | 1.46 | 1.2 | -0.886 | 3.81 | 0.242 | 1 |
| CD9 | 1.54 | 1.27 | -0.946 | 4.02 | 0.245 | 1 |
| ELK1 | 0.684 | 0.566 | -0.425 | 1.79 | 0.247 | 1 |
| ISG15 | 0.726 | 0.602 | -0.454 | 1.9 | 0.248 | 1 |
| TYK2 | 1.23 | 1.02 | -0.772 | 3.23 | 0.248 | 1 |
| MARCO | -1.57 | 1.31 | -4.14 | 1.01 | 0.252 | 1 |
| ALCAM | 0.74 | 0.62 | -0.475 | 1.95 | 0.252 | 1 |
| IFITM1 | 0.639 | 0.538 | -0.416 | 1.69 | 0.255 | 1 |
| MAP3K5 | 0.449 | 0.381 | -0.297 | 1.2 | 0.258 | 1 |
| ENG | 0.466 | 0.395 | -0.309 | 1.24 | 0.258 | 1 |
| ANXA1 | 0.4 | 0.341 | -0.268 | 1.07 | 0.26 | 1 |
| MAPK1 | 1.25 | 1.07 | -0.84 | 3.35 | 0.26 | 1 |
| IL15RA | 0.742 | 0.633 | -0.5 | 1.98 | 0.261 | 1 |
| CD58 | 1.49 | 1.28 | -1.01 | 3.99 | 0.262 | 1 |
| IFIT1 | 0.579 | 0.507 | -0.414 | 1.57 | 0.272 | 1 |
| IFNAR2 | 0.192 | 0.169 | -0.139 | 0.523 | 0.275 | 1 |
| IFI35 | 0.603 | 0.532 | -0.439 | 1.65 | 0.275 | 1 |
| CCL8 | -0.441 | 0.39 | -1.2 | 0.322 | 0.276 | 1 |
| STAT1 | 0.584 | 0.519 | -0.433 | 1.6 | 0.279 | 1 |
| SERPING1 | 0.525 | 0.468 | -0.393 | 1.44 | 0.281 | 1 |
| CD22 | 0.785 | 0.701 | -0.589 | 2.16 | 0.282 | 1 |
| MICB | 1.1 | 0.991 | -0.846 | 3.04 | 0.287 | 1 |
| RPS6 | 0.422 | 0.382 | -0.327 | 1.17 | 0.288 | 1 |
| FCER1A | -0.835 | 0.763 | -2.33 | 0.661 | 0.292 | 1 |
| AXL | 0.457 | 0.418 | -0.363 | 1.28 | 0.293 | 1 |
| PYCARD | 0.651 | 0.599 | -0.523 | 1.82 | 0.296 | 1 |
| NUP107 | 0.268 | 0.249 | -0.22 | 0.756 | 0.3 | 1 |
| IRF7 | 0.508 | 0.473 | -0.419 | 1.44 | 0.301 | 1 |
| HLA-E | 0.368 | 0.347 | -0.312 | 1.05 | 0.306 | 1 |
| NFKBIA | 1.09 | 1.03 | -0.933 | 3.11 | 0.309 | 1 |
| ENTPD1 | 0.689 | 0.658 | -0.601 | 1.98 | 0.313 | 1 |
| TNF | 0.723 | 0.691 | -0.632 | 2.08 | 0.313 | 1 |
| MYD88 | 0.567 | 0.545 | -0.502 | 1.64 | 0.316 | 1 |
| TARP | 0.804 | 0.775 | -0.714 | 2.32 | 0.317 | 1 |
| IKBKE | 0.57 | 0.552 | -0.511 | 1.65 | 0.319 | 1 |
| CD96 | -0.562 | 0.551 | -1.64 | 0.517 | 0.325 | 1 |
| MCAM | 0.395 | 0.391 | -0.371 | 1.16 | 0.329 | 1 |
| BIRC5 | -0.307 | 0.305 | -0.905 | 0.29 | 0.331 | 1 |
| SBNO2 | 0.248 | 0.247 | -0.236 | 0.733 | 0.332 | 1 |
| IFNGR1 | 1.17 | 1.16 | -1.11 | 3.44 | 0.332 | 1 |
| FCGR2B | -0.71 | 0.709 | -2.1 | 0.679 | 0.333 | 1 |
| NFKB1 | 0.624 | 0.625 | -0.602 | 1.85 | 0.335 | 1 |
| MEF2C | 0.616 | 0.62 | -0.6 | 1.83 | 0.338 | 1 |
| BCL6 | 0.256 | 0.259 | -0.253 | 0.764 | 0.341 | 1 |
| TGFB1 | 0.407 | 0.414 | -0.404 | 1.22 | 0.342 | 1 |
| GPI | 1.09 | 1.12 | -1.1 | 3.28 | 0.346 | 1 |
| CD80 | 0.687 | 0.709 | -0.703 | 2.08 | 0.349 | 1 |
| GZMH | 0.636 | 0.658 | -0.653 | 1.92 | 0.35 | 1 |
| TFEB | 0.56 | 0.583 | -0.582 | 1.7 | 0.353 | 1 |
| PSMB8 | 0.422 | 0.445 | -0.45 | 1.29 | 0.359 | 1 |
| CD163 | 0.533 | 0.569 | -0.583 | 1.65 | 0.365 | 1 |
| PRKCD | 0.537 | 0.58 | -0.6 | 1.67 | 0.37 | 1 |
| TCF7 | 1.17 | 1.27 | -1.31 | 3.65 | 0.371 | 1 |
| TNFSF15 | 0.576 | 0.624 | -0.648 | 1.8 | 0.372 | 1 |
| CXCR2 | 0.762 | 0.827 | -0.859 | 2.38 | 0.372 | 1 |
| CXCR4 | -0.596 | 0.648 | -1.86 | 0.674 | 0.373 | 1 |
| ATF1 | 0.804 | 0.887 | -0.934 | 2.54 | 0.38 | 1 |
| SMPD3 | 1.21 | 1.34 | -1.42 | 3.83 | 0.382 | 1 |
| PSMB10 | 0.415 | 0.463 | -0.492 | 1.32 | 0.385 | 1 |
| TANK | 0.427 | 0.477 | -0.509 | 1.36 | 0.386 | 1 |
| CFP | -0.507 | 0.569 | -1.62 | 0.609 | 0.388 | 1 |
| CLEC4A | 0.325 | 0.365 | -0.39 | 1.04 | 0.388 | 1 |
| DOCK9 | 0.432 | 0.487 | -0.522 | 1.39 | 0.39 | 1 |
| HMGB1 | 0.258 | 0.291 | -0.313 | 0.828 | 0.391 | 1 |
| IL1RN | -0.59 | 0.668 | -1.9 | 0.719 | 0.392 | 1 |
| CTSW | 0.643 | 0.73 | -0.789 | 2.07 | 0.394 | 1 |
| NT5E | -0.462 | 0.531 | -1.5 | 0.579 | 0.399 | 1 |
| CCL3L1 | 0.683 | 0.791 | -0.866 | 2.23 | 0.402 | 1 |
| C1R | 0.36 | 0.417 | -0.458 | 1.18 | 0.403 | 1 |
| ADA | -0.255 | 0.309 | -0.86 | 0.35 | 0.423 | 1 |
| TAP2 | 0.267 | 0.324 | -0.369 | 0.903 | 0.425 | 1 |
| PSMB9 | 0.218 | 0.267 | -0.305 | 0.74 | 0.427 | 1 |
| TXNIP | 0.273 | 0.335 | -0.383 | 0.929 | 0.428 | 1 |
| CD48 | 0.552 | 0.676 | -0.774 | 1.88 | 0.428 | 1 |
| REL | 0.363 | 0.457 | -0.533 | 1.26 | 0.441 | 1 |
| LGALS3 | 0.284 | 0.359 | -0.419 | 0.987 | 0.442 | 1 |
| NFKB2 | 0.252 | 0.32 | -0.374 | 0.879 | 0.443 | 1 |
| CD81 | 0.303 | 0.387 | -0.456 | 1.06 | 0.447 | 1 |
| ANP32B | 0.341 | 0.442 | -0.526 | 1.21 | 0.453 | 1 |
| SIGIRR | 0.291 | 0.383 | -0.459 | 1.04 | 0.459 | 1 |
| CD247 | 0.595 | 0.794 | -0.96 | 2.15 | 0.466 | 1 |
| CLEC7A | -0.513 | 0.686 | -1.86 | 0.831 | 0.467 | 1 |
| CARD9 | 0.497 | 0.678 | -0.833 | 1.83 | 0.476 | 1 |
| ITGAX | -0.43 | 0.591 | -1.59 | 0.728 | 0.479 | 1 |
| HAVCR2 | -0.219 | 0.3 | -0.808 | 0.37 | 0.479 | 1 |
| CD4 | 0.323 | 0.446 | -0.551 | 1.2 | 0.48 | 1 |
| APOE | 0.56 | 0.771 | -0.952 | 2.07 | 0.48 | 1 |
| IFITM2 | 0.262 | 0.365 | -0.453 | 0.976 | 0.485 | 1 |
| TLR4 | 0.401 | 0.56 | -0.696 | 1.5 | 0.485 | 1 |
| C1QA | 0.511 | 0.713 | -0.886 | 1.91 | 0.485 | 1 |
| NRP1 | 0.31 | 0.434 | -0.54 | 1.16 | 0.486 | 1 |
| CR1 | -0.531 | 0.744 | -1.99 | 0.927 | 0.487 | 1 |
| IFNAR1 | 0.16 | 0.227 | -0.284 | 0.604 | 0.492 | 1 |
| CD3D | 0.554 | 0.787 | -0.987 | 2.1 | 0.492 | 1 |
| IL6R | 0.429 | 0.614 | -0.774 | 1.63 | 0.496 | 1 |
| TNFRSF10B | -0.113 | 0.163 | -0.433 | 0.206 | 0.498 | 1 |
| THY1 | 0.231 | 0.334 | -0.425 | 0.886 | 0.502 | 1 |
| HLA-DRB3 | 0.389 | 0.569 | -0.725 | 1.5 | 0.505 | 1 |
| MUC1 | 0.325 | 0.478 | -0.611 | 1.26 | 0.508 | 1 |
| BCL10 | 0.345 | 0.515 | -0.664 | 1.36 | 0.514 | 1 |
| GZMK | 0.543 | 0.81 | -1.05 | 2.13 | 0.514 | 1 |
| C1QB | 0.443 | 0.665 | -0.861 | 1.75 | 0.516 | 1 |
| FCGR1A | 0.504 | 0.756 | -0.978 | 1.99 | 0.516 | 1 |
| IL2RA | 0.624 | 0.937 | -1.21 | 2.46 | 0.516 | 1 |
| BAX | 0.379 | 0.572 | -0.742 | 1.5 | 0.519 | 1 |
| SLAMF7 | 0.485 | 0.741 | -0.967 | 1.94 | 0.523 | 1 |
| EGR1 | 0.397 | 0.612 | -0.803 | 1.6 | 0.528 | 1 |
| GZMA | 0.543 | 0.838 | -1.1 | 2.19 | 0.528 | 1 |
| ITGB1 | 0.278 | 0.433 | -0.571 | 1.13 | 0.531 | 1 |
| CD53 | 0.834 | 1.3 | -1.72 | 3.38 | 0.532 | 1 |
| IRAK1 | 0.101 | 0.16 | -0.211 | 0.414 | 0.536 | 1 |
| TAPBP | 0.17 | 0.268 | -0.355 | 0.694 | 0.536 | 1 |
| IL21R | 0.434 | 0.689 | -0.916 | 1.78 | 0.538 | 1 |
| KLRC1 | 0.29 | 0.461 | -0.614 | 1.19 | 0.539 | 1 |
| S100A8 | -0.54 | 0.86 | -2.23 | 1.15 | 0.54 | 1 |
| POU2F2 | -0.38 | 0.609 | -1.57 | 0.813 | 0.542 | 1 |
| TNFSF10 | 0.392 | 0.628 | -0.838 | 1.62 | 0.542 | 1 |
| IL10RA | 0.346 | 0.562 | -0.755 | 1.45 | 0.547 | 1 |
| CD40 | 0.223 | 0.362 | -0.486 | 0.932 | 0.548 | 1 |
| CD74 | 0.393 | 0.638 | -0.857 | 1.64 | 0.548 | 1 |
| TNFRSF9 | 0.421 | 0.687 | -0.925 | 1.77 | 0.55 | 1 |
| CSF2RB | 0.336 | 0.55 | -0.743 | 1.41 | 0.551 | 1 |
| BLNK | 0.511 | 0.836 | -1.13 | 2.15 | 0.551 | 1 |
| IL1RAP | 0.64 | 1.06 | -1.43 | 2.71 | 0.554 | 1 |
| ATG7 | 0.339 | 0.567 | -0.772 | 1.45 | 0.559 | 1 |
| CFI | 0.256 | 0.432 | -0.59 | 1.1 | 0.562 | 1 |
| HLA-A | 0.2 | 0.339 | -0.465 | 0.865 | 0.564 | 1 |
| TNFAIP3 | 0.306 | 0.521 | -0.716 | 1.33 | 0.566 | 1 |
| LAIR2 | 0.493 | 0.84 | -1.15 | 2.14 | 0.566 | 1 |
| CD36 | -0.347 | 0.598 | -1.52 | 0.824 | 0.571 | 1 |
| IL16 | 0.375 | 0.672 | -0.942 | 1.69 | 0.585 | 1 |
| TNFSF11 | -0.251 | 0.45 | -1.13 | 0.63 | 0.586 | 1 |
| CD68 | 0.339 | 0.614 | -0.864 | 1.54 | 0.59 | 1 |
| CCR2 | 0.41 | 0.744 | -1.05 | 1.87 | 0.59 | 1 |
| C2 | 0.281 | 0.513 | -0.724 | 1.29 | 0.592 | 1 |
| IL18 | 0.188 | 0.343 | -0.485 | 0.86 | 0.593 | 1 |
| ITGAL | 0.354 | 0.648 | -0.916 | 1.62 | 0.593 | 1 |
| TLR5 | 0.369 | 0.676 | -0.955 | 1.69 | 0.593 | 1 |
| IKBKB | 0.243 | 0.449 | -0.638 | 1.12 | 0.598 | 1 |
| BST1 | 0.282 | 0.533 | -0.763 | 1.33 | 0.605 | 1 |
| FCGR2A | -0.233 | 0.444 | -1.1 | 0.637 | 0.607 | 1 |
| HLA-DMB | 0.404 | 0.797 | -1.16 | 1.97 | 0.62 | 1 |
| CD37 | 0.403 | 0.799 | -1.16 | 1.97 | 0.622 | 1 |
| IL1R1 | 0.278 | 0.552 | -0.805 | 1.36 | 0.623 | 1 |
| CD44 | 0.215 | 0.43 | -0.629 | 1.06 | 0.626 | 1 |
| CD86 | 0.33 | 0.662 | -0.968 | 1.63 | 0.626 | 1 |
| CD274 | -0.325 | 0.657 | -1.61 | 0.962 | 0.628 | 1 |
| ST6GAL1 | 0.385 | 0.781 | -1.15 | 1.92 | 0.63 | 1 |
| CYLD | 0.105 | 0.213 | -0.313 | 0.522 | 0.631 | 1 |
| FEZ1 | 0.252 | 0.512 | -0.752 | 1.26 | 0.631 | 1 |
| ULBP2 | -0.222 | 0.459 | -1.12 | 0.677 | 0.635 | 1 |
| TNFSF8 | -0.273 | 0.564 | -1.38 | 0.832 | 0.636 | 1 |
| VCAM1 | 0.355 | 0.736 | -1.09 | 1.8 | 0.637 | 1 |
| CCR5 | -0.391 | 0.813 | -1.98 | 1.2 | 0.638 | 1 |
| FN1 | -0.301 | 0.626 | -1.53 | 0.926 | 0.638 | 1 |
| SH2B2 | 0.238 | 0.502 | -0.746 | 1.22 | 0.642 | 1 |
| LY86 | 0.376 | 0.797 | -1.19 | 1.94 | 0.644 | 1 |
| MAP3K1 | -0.134 | 0.284 | -0.691 | 0.423 | 0.645 | 1 |
| TNFSF13B | 0.307 | 0.65 | -0.968 | 1.58 | 0.645 | 1 |
| AMICA1 | -0.212 | 0.456 | -1.11 | 0.681 | 0.648 | 1 |
| TLR2 | -0.201 | 0.443 | -1.07 | 0.667 | 0.657 | 1 |
| NFATC2 | 0.271 | 0.6 | -0.905 | 1.45 | 0.658 | 1 |
| FCER2 | -0.339 | 0.757 | -1.82 | 1.15 | 0.661 | 1 |
| FCGR3A | -0.286 | 0.648 | -1.56 | 0.984 | 0.665 | 1 |
| SELL | 0.272 | 0.617 | -0.938 | 1.48 | 0.667 | 1 |
| DPP4 | 0.3 | 0.682 | -1.04 | 1.64 | 0.667 | 1 |
| CD83 | -0.203 | 0.463 | -1.11 | 0.705 | 0.668 | 1 |
| IL7R | -0.299 | 0.692 | -1.66 | 1.06 | 0.672 | 1 |
| CCL2 | -0.281 | 0.66 | -1.57 | 1.01 | 0.677 | 1 |
| CD14 | -0.25 | 0.588 | -1.4 | 0.903 | 0.677 | 1 |
| SIGLEC1 | 0.345 | 0.826 | -1.28 | 1.96 | 0.683 | 1 |
| PLA2G6 | -0.138 | 0.331 | -0.785 | 0.51 | 0.684 | 1 |
| SELPLG | 0.32 | 0.773 | -1.2 | 1.84 | 0.685 | 1 |
| LCP1 | 0.148 | 0.358 | -0.555 | 0.85 | 0.686 | 1 |
| IRF3 | 0.079 | 0.192 | -0.298 | 0.456 | 0.688 | 1 |
| PIK3CD | 0.307 | 0.751 | -1.16 | 1.78 | 0.688 | 1 |
| CD28 | 0.308 | 0.752 | -1.17 | 1.78 | 0.689 | 1 |
| ITGB4 | 0.25 | 0.618 | -0.961 | 1.46 | 0.692 | 1 |
| PSMB7 | -0.0655 | 0.165 | -0.388 | 0.257 | 0.697 | 1 |
| THBD | -0.219 | 0.556 | -1.31 | 0.871 | 0.7 | 1 |
| NFATC3 | 0.107 | 0.272 | -0.426 | 0.64 | 0.701 | 1 |
| CDKN1A | 0.15 | 0.41 | -0.654 | 0.954 | 0.72 | 1 |
| BST2 | 0.215 | 0.59 | -0.942 | 1.37 | 0.721 | 1 |
| TFRC | -0.254 | 0.705 | -1.64 | 1.13 | 0.724 | 1 |
| PDCD1LG2 | 0.214 | 0.615 | -0.991 | 1.42 | 0.733 | 1 |
| ICOSLG | 0.224 | 0.649 | -1.05 | 1.5 | 0.735 | 1 |
| HLA-DQB1 | 0.778 | 2.26 | -3.66 | 5.22 | 0.736 | 1 |
| DUSP6 | 0.14 | 0.409 | -0.662 | 0.942 | 0.737 | 1 |
| ITGA4 | 0.219 | 0.65 | -1.06 | 1.49 | 0.742 | 1 |
| HLA-DMA | 0.232 | 0.692 | -1.13 | 1.59 | 0.743 | 1 |
| FOS | 0.206 | 0.624 | -1.02 | 1.43 | 0.746 | 1 |
| KLRD1 | -0.202 | 0.633 | -1.44 | 1.04 | 0.755 | 1 |
| CXCR3 | -0.226 | 0.712 | -1.62 | 1.17 | 0.756 | 1 |
| ITGA5 | 0.107 | 0.343 | -0.566 | 0.78 | 0.759 | 1 |
| LTB | -0.187 | 0.607 | -1.38 | 1 | 0.763 | 1 |
| STAT4 | 0.145 | 0.477 | -0.79 | 1.08 | 0.765 | 1 |
| FADD | -0.0666 | 0.219 | -0.496 | 0.363 | 0.766 | 1 |
| CD59 | 0.103 | 0.344 | -0.57 | 0.777 | 0.768 | 1 |
| CASP1 | 0.185 | 0.619 | -1.03 | 1.4 | 0.769 | 1 |
| LILRA5 | 0.204 | 0.699 | -1.17 | 1.57 | 0.775 | 1 |
| EBI3 | -0.198 | 0.691 | -1.55 | 1.16 | 0.779 | 1 |
| HLA-DQA1 | 0.742 | 2.65 | -4.45 | 5.93 | 0.784 | 1 |
| CFB | 0.14 | 0.506 | -0.852 | 1.13 | 0.786 | 1 |
| MSR1 | 0.22 | 0.799 | -1.35 | 1.78 | 0.787 | 1 |
| ITGA6 | 0.181 | 0.671 | -1.14 | 1.5 | 0.792 | 1 |
| BID | 0.13 | 0.491 | -0.831 | 1.09 | 0.794 | 1 |
| MIF | -0.0959 | 0.367 | -0.814 | 0.622 | 0.797 | 1 |
| SPP1 | -0.147 | 0.571 | -1.27 | 0.971 | 0.8 | 1 |
| ZNF205 | 0.0611 | 0.237 | -0.404 | 0.526 | 0.8 | 1 |
| HLA-DPA1 | 0.172 | 0.676 | -1.15 | 1.5 | 0.802 | 1 |
| GTF3C1 | 0.12 | 0.474 | -0.81 | 1.05 | 0.803 | 1 |
| CARD11 | -0.115 | 0.452 | -1 | 0.772 | 0.804 | 1 |
| HLA-DPB1 | 0.177 | 0.702 | -1.2 | 1.55 | 0.804 | 1 |
| FCER1G | 0.125 | 0.498 | -0.851 | 1.1 | 0.805 | 1 |
| MAP4K2 | -0.0424 | 0.172 | -0.379 | 0.294 | 0.809 | 1 |
| CSF3R | -0.182 | 0.742 | -1.64 | 1.27 | 0.81 | 1 |
| NLRP3 | 0.192 | 0.79 | -1.36 | 1.74 | 0.811 | 1 |
| CTSL | -0.11 | 0.457 | -1 | 0.785 | 0.814 | 1 |
| TNFSF13 | -0.115 | 0.482 | -1.06 | 0.829 | 0.815 | 1 |
| HLA-C | -0.0847 | 0.37 | -0.809 | 0.64 | 0.822 | 1 |
| IRF5 | 0.14 | 0.626 | -1.09 | 1.37 | 0.826 | 1 |
| CFD | -0.152 | 0.689 | -1.5 | 1.2 | 0.828 | 1 |
| TLR1 | -0.0944 | 0.434 | -0.946 | 0.757 | 0.831 | 1 |
| PLAU | -0.0984 | 0.459 | -0.998 | 0.801 | 0.833 | 1 |
| CCL4 | -0.101 | 0.482 | -1.05 | 0.843 | 0.837 | 1 |
| TREM2 | 0.169 | 0.816 | -1.43 | 1.77 | 0.839 | 1 |
| IL1R2 | 0.207 | 1.01 | -1.78 | 2.19 | 0.841 | 1 |
| CTSS | 0.138 | 0.69 | -1.21 | 1.49 | 0.845 | 1 |
| CXCL14 | 0.134 | 0.7 | -1.24 | 1.51 | 0.851 | 1 |
| TNFRSF14 | 0.107 | 0.566 | -1 | 1.22 | 0.853 | 1 |
| TNFRSF12A | -0.0772 | 0.417 | -0.894 | 0.739 | 0.856 | 1 |
| LILRB2 | -0.0953 | 0.551 | -1.17 | 0.984 | 0.865 | 1 |
| PTPRC | 0.0751 | 0.476 | -0.858 | 1.01 | 0.877 | 1 |
| CCL5 | -0.0578 | 0.371 | -0.785 | 0.67 | 0.878 | 1 |
| LILRB3 | 0.0912 | 0.584 | -1.05 | 1.24 | 0.878 | 1 |
| TNFRSF10C | 0.0677 | 0.441 | -0.797 | 0.933 | 0.88 | 1 |
| CCL13 | 0.147 | 0.97 | -1.75 | 2.05 | 0.881 | 1 |
| HLA-DRA | -0.0918 | 0.609 | -1.29 | 1.1 | 0.882 | 1 |
| IL2RG | -0.0697 | 0.475 | -1 | 0.862 | 0.885 | 1 |
| HLA-B | 0.0597 | 0.411 | -0.746 | 0.866 | 0.887 | 1 |
| CXCR1 | 0.0839 | 0.601 | -1.09 | 1.26 | 0.891 | 1 |
| LAMP3 | -0.108 | 0.788 | -1.65 | 1.44 | 0.892 | 1 |
| KLRK1 | 0.0956 | 0.719 | -1.31 | 1.5 | 0.896 | 1 |
| CASP3 | -0.0758 | 0.584 | -1.22 | 1.07 | 0.899 | 1 |
| HCK | -0.0544 | 0.42 | -0.877 | 0.768 | 0.899 | 1 |
| MRC1 | -0.0865 | 0.676 | -1.41 | 1.24 | 0.9 | 1 |
| MERTK | 0.0538 | 0.449 | -0.827 | 0.934 | 0.906 | 1 |
| PRF1 | 0.102 | 0.875 | -1.61 | 1.82 | 0.909 | 1 |
| RUNX1 | -0.0311 | 0.279 | -0.579 | 0.516 | 0.913 | 1 |
| PVR | 0.037 | 0.338 | -0.626 | 0.7 | 0.914 | 1 |
| CCR1 | 0.0567 | 0.537 | -0.996 | 1.11 | 0.917 | 1 |
| SYK | 0.0586 | 0.568 | -1.06 | 1.17 | 0.919 | 1 |
| LILRB1 | -0.0606 | 0.62 | -1.28 | 1.15 | 0.923 | 1 |
| ISG20 | 0.0414 | 0.424 | -0.79 | 0.873 | 0.924 | 1 |
| CXCL16 | 0.0491 | 0.525 | -0.98 | 1.08 | 0.927 | 1 |
| ICAM3 | 0.0212 | 0.235 | -0.439 | 0.481 | 0.929 | 1 |
| INPP5D | 0.0658 | 0.725 | -1.36 | 1.49 | 0.929 | 1 |
| PDGFC | -0.064 | 0.727 | -1.49 | 1.36 | 0.931 | 1 |
| IRF8 | -0.0671 | 0.83 | -1.69 | 1.56 | 0.937 | 1 |
| ITGA2 | -0.0434 | 0.596 | -1.21 | 1.12 | 0.943 | 1 |
| CXCL5 | 0.106 | 1.55 | -2.93 | 3.14 | 0.946 | 1 |
| LY9 | -0.0498 | 0.737 | -1.49 | 1.39 | 0.947 | 1 |
| SLAMF6 | -0.0474 | 0.698 | -1.42 | 1.32 | 0.947 | 1 |
| ITGB2 | 0.0357 | 0.526 | -0.995 | 1.07 | 0.947 | 1 |
| DUSP4 | 0.0437 | 0.758 | -1.44 | 1.53 | 0.955 | 1 |
| ADORA2A | -0.0254 | 0.466 | -0.938 | 0.888 | 0.957 | 1 |
| C3AR1 | 0.0394 | 0.731 | -1.39 | 1.47 | 0.958 | 1 |
| CKLF | -0.0193 | 0.369 | -0.743 | 0.704 | 0.959 | 1 |
| ITK | 0.0351 | 0.678 | -1.29 | 1.36 | 0.959 | 1 |
| MFGE8 | 0.0384 | 0.736 | -1.4 | 1.48 | 0.959 | 1 |
| CD209 | 0.038 | 0.736 | -1.4 | 1.48 | 0.96 | 1 |
| TLR9 | -0.0245 | 0.559 | -1.12 | 1.07 | 0.966 | 1 |
| ITGAM | 0.027 | 0.723 | -1.39 | 1.44 | 0.971 | 1 |
| CYBB | 0.0277 | 0.768 | -1.48 | 1.53 | 0.972 | 1 |
| MX1 | 0.0134 | 0.431 | -0.832 | 0.859 | 0.976 | 1 |
| CTSH | 0.0134 | 0.457 | -0.882 | 0.909 | 0.977 | 1 |
| F13A1 | -0.0196 | 0.72 | -1.43 | 1.39 | 0.979 | 1 |
| CD180 | 0.017 | 0.757 | -1.47 | 1.5 | 0.982 | 1 |
| THBS1 | 0.00961 | 0.506 | -0.983 | 1 | 0.985 | 1 |
| LYN | -0.0113 | 0.637 | -1.26 | 1.24 | 0.986 | 1 |
| TLR7 | 0.00892 | 0.82 | -1.6 | 1.62 | 0.991 | 1 |
| TNFRSF8 | -0.00601 | 0.648 | -1.28 | 1.26 | 0.993 | 1 |
| LIF | 0.00143 | 0.588 | -1.15 | 1.15 | 0.998 | 1 |
| TLR6 | 0.00155 | 0.651 | -1.27 | 1.28 | 0.998 | 1 |
| TGFB2 | -0.00012 | 0.42 | -0.823 | 0.822 | 1 | 1 |

Table S9. Missingness of proteins by sample used in the proteomic analysis.

| Sample ID | Sample 7 | Sample 12 | Sample 8 | Sample 6 | Sample 9 | Sample 10 |
| --- | --- | --- | --- | --- | --- | --- |
| No. Proteins Observed | *5604* | *5546* | *3154* | *5130* | *5475* | *3165* |
| Observed (%) | *93.57%* | *92.60%* | *52.66%* | *85.66%* | *91.42%* | *52.85%* |
| Missingness (%) | *6.43%* | *7.40%* | *47.34%* | *14.34%* | *8.58%* | *47.15%* |

4 References

1.	Boriani S, Bandiera S, Casadei R, et al. Giant cell tumor of the mobile spine: A review of 49 cases. *Spine (Phila Pa 1976)*. 2012;37(1). doi:10.1097/BRS.0b013e3182233ccd

2.	Birch R, Eltit F, Xie D, et al. Denosumab treatment of giant cell tumors in the spine induces woven bone formation. *JBMR Plus*. 25AD;9(6). doi:10.1093/jbmrpl/ziaf063
